# Tandem duplication upstream of *HSF5* associates with transcript remodeling and triple-comb morphology in chickens

**DOI:** 10.1016/j.isci.2026.117678

**Published:** 2026-09-28

**Authors:** Xun-He Huang, Zhi-Feng Zhang, Zhi-Peng Zhong, Zhuo-Xian Weng, Yong-Jie Xu, Guo-Hao Zhong, Wei-Na Li, Qing Wang, Bing-Wang Du, Min-Sheng Peng, Cheng Ma

**Affiliations:** 1 Guangdong Provincial Key Laboratory of Conservation and Precision Utilization of Characteristic Agricultural Resources in Mountainous Areas, Guangdong Innovation Centre for Science and Technology of Wuhua Yellow Chicken, School of Life Sciences, Jiaying University, Meizhou 514015, China; 2 State Key Laboratory of Genetic Evolution & Animal Models and Yunnan Key Laboratory of Molecular Biology of Domestic Animals, Kunming Institute of Zoology, Chinese Academy of Sciences, Kunming 650201, China; 3 Kunming College of Life Science, University of Chinese Academy of Sciences, Kunming 650204, China; 4 Department of Medical Biochemistry and Microbiology, Uppsala University, 75123 Uppsala, Sweden

**Keywords:** chicken, triple comb, tandem duplication, *HSF5*, transcript remodeling

## Abstract

The genetic basis of the triple comb in chickens remains unknown. Here, we integrated population genomics, genome-wide association studies, structural variant analysis, long-read sequencing, and embryonic transcriptomics in Wuhua Yellow chickens. We identified a 15.7-kb tandem duplication upstream of *HSF5* on chromosome 19 as the primary candidate. At embryonic day 8, overall *HSF5* expression was unchanged, yet we observed localized exon upregulation within the duplicated region, a dosage-dependent expression gradient, and extensive non-canonical splicing. Public epigenomic data further indicate baseline enhancer activity in this interval. Copy number correlated positively with comb prong number (*R*^2^ = 0.45, adjusted *p* = 9.26 × 10^−7^). Exploratory fertility assessments revealed a lower fertilization rate in triple-comb crosses, suggesting a potential pleiotropic cost. These findings strongly implicate *HSF5* as a key candidate and demonstrate that a tandem duplication associates with phenotypic innovation through transcript remodeling rather than simple gene misexpression.

## Highlights

• A 15.7-kb tandem duplication upstream of *HSF5* associates with triple-comb morphology
• Localized exon upregulation and non-canonical splicing suggest transcript remodeling
• *HSF5* copy number correlates with comb prong number, suggesting dosage dependence
• Population genetics support selection on the *HSF5* duplication haplotype

## Graphical abstract

**Figure:**
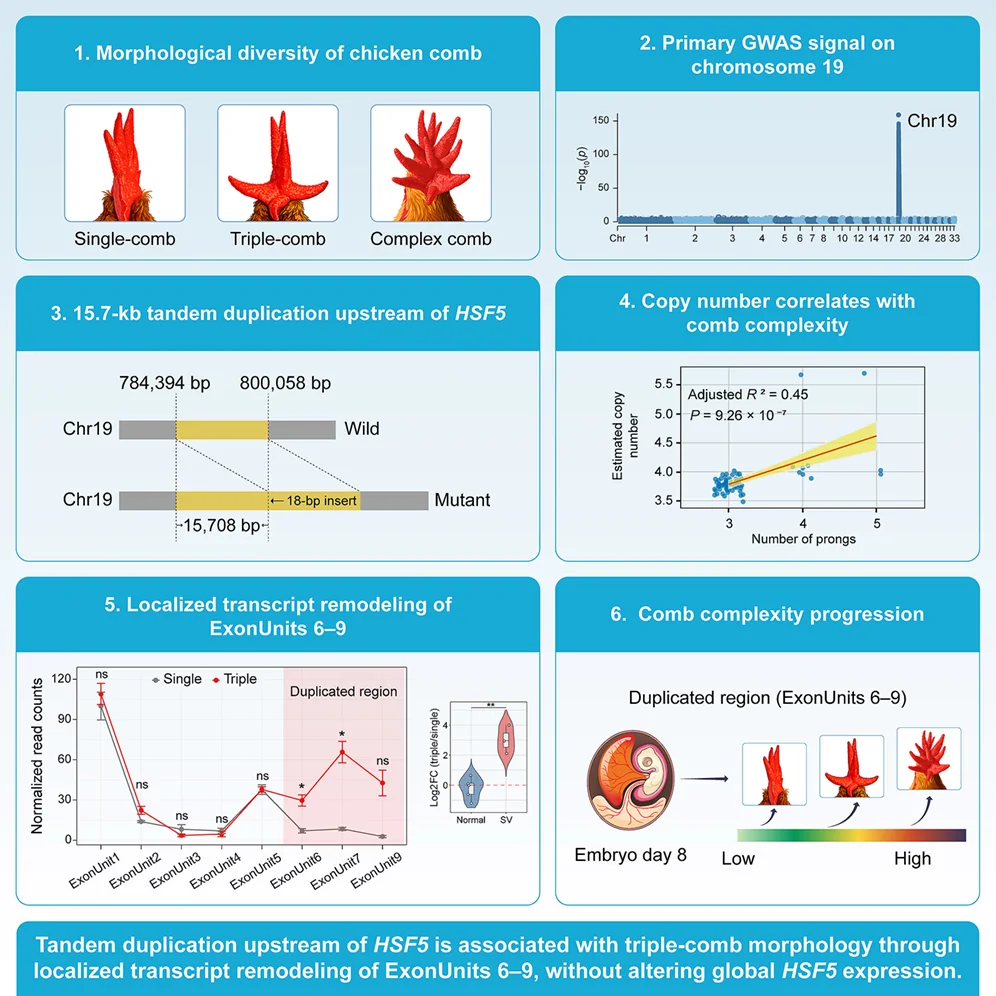

## Teaser

Bioinformatics; Evolutionary biology; Genomics

## Introduction

The chicken comb, a defining integumentary appendage of *Gallus gallus*, plays critical roles in thermoregulation and social signaling and serves as a primary visual marker of breed identity in poultry production.^1^^,^^2^^,^^3^ This functional importance is reflected in the remarkable diversity of comb morphologies across domestic breeds, which has provided foundational models for genetic studies ranging from Mendelian inheritance to epistasis.^4^^,^^5^ Over the past two decades, research has converged on a clear paradigm: major classical comb types, including Pea, Rose, and Duplex, are predominantly caused by structural genomic rearrangements (e.g., duplications and inversions) that alter the spatiotemporal expression of key developmental regulators, such as *SOX5*, *MNR2*, and *EOMES,* during embryogenesis.^6^^,^^7^^,^^8^^,^^9^^,^^10^ This highlights the pivotal role of *cis*-regulatory evolution in driving rapid phenotypic diversification during domestication.^11^

In contrast, the triple comb, a distinct phenotype characterized by pronounced posterior bifurcation, remains genetically unresolved. Within this morphological continuum, the standard “triple comb” features a distinct posterior split into three ridges, whereas the “supernumerary-prong comb” represents a more complex variant with additional secondary prongs branching from these ridges. The elaborate “deer-antler comb” observed in Hetian chickens represents the extreme manifestation of this spectrum, exhibiting advanced, staghorn-like multi-branching.^12^ Early studies documented its occurrence and suggested a complex, potentially polygenic inheritance.^13^^,^^14^^,^^15^ Subsequent research in indigenous Chinese chickens has produced successive yet sometimes conflicting findings. Initial studies on Qingyuan Partridge chickens, based on hybrid crosses, described segregation patterns consistent with polygenic inheritance but did not identify specific causative loci.^16^ A later study in Hetian chickens identified a 15.7-kb duplication upstream of the gene encoding heat shock transcription factor 5 (*HSF5*) that is strongly associated with the pronounced “deer-antler” comb phenotype.^17^ However, that study was limited by sample size and focused primarily on gene expression, leaving the structural architecture of the duplication, its dosage effects, and its impact on transcript diversity unresolved. Notably, a recent genome-wide association study (GWAS) in Qingyuan Partridge chickens identified a significant locus on chromosome 13 encompassing the calcium/calmodulin-dependent protein kinase II alpha (*CAMK2A*) gene, with no association detected at the *HSF*5 locus.^18^

The discrepant findings across breeds point to potential genetic heterogeneity or context-dependent effects of the *HSF5* duplication, leaving a critical gap in our understanding of comb morphogenesis. The Wuhua Yellow chicken provides a valuable independent model to address these questions, as it exhibits a continuous spectrum of comb morphogenesis, ranging from the single-comb to the standard triple-comb and more complex variants with supernumerary prongs (Figure 1). Previous genomic studies of this breed have identified selection signatures associated with traits such as feather color,^19^^,^^20^^,^^21^^,^^22^ indicating that the Wuhua Yellow chicken harbors functionally relevant variation and establishing it as a well-characterized resource for studying morphological diversity.

**Figure 1:**
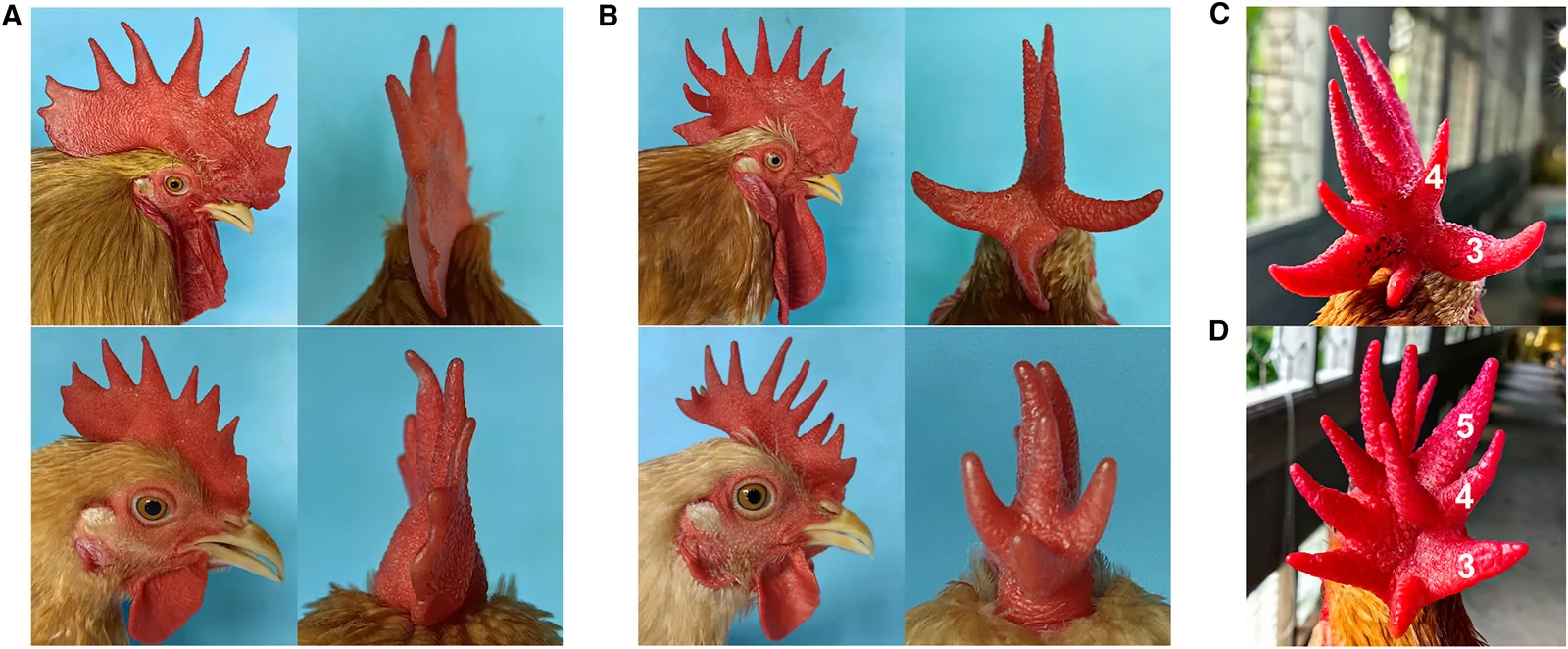
Phenotypic diversity of comb morphology in Wuhua Yellow chickens (A) Single-comb morphology, characterized by an undivided posterior comb. For each sex, the phenotype is shown in lateral and caudal-to-rostral views (upper row, roosters; lower row, hens; left column, lateral view; right column, caudal-to-rostral view). (B) Standard triple-comb morphology, defined by a clear posterior bifurcation, displayed in the same layout as in (A). (C and D) Complex comb morphologies with supernumerary prongs, with prong numbers annotated on each image (C: 3–4; D: 3–5). To ensure consistency, prongs were counted from a caudal-to-rostral perspective, where “3” represents the standard three-ridged base, and “4” and “5” denote secondary prongs branching from the lateral or posterior margins. These elaborate forms represent extreme variants along the bifurcation spectrum and are phenotypically continuous with the standard triple comb. In this study, “triple comb” refers specifically to the standard bifurcated morphology shown in (B), which serves as the primary focus of our genetic and functional analyses.

In the present study, we aimed to assess whether the *HSF5* duplication is consistently associated with the triple-comb phenotype across lineages and to investigate the molecular mechanism associated with this structural variant (SV). Here, we used “triple comb” to specifically refer to the standard bifurcated morphology (Figure 1B) and focused our analyses on this form in Wuhua Yellow chickens. Furthermore, we leveraged read-depth analysis to examine how SV copy-number variations (CNVs) correlate with prong numbers, presenting these dosage-effect findings as supportive evidence for phenotypic scaling. Finally, we examined whether this duplication alters *HSF5* transcript architecture during embryonic development and evaluated its contribution to the broader paradigms of SV-driven morphological diversification in chickens.

## Results

### Genome-wide mapping identifies a major triple-comb locus on chromosome 19

To define the genetic basis of the triple-comb morphology, we performed whole-genome resequencing of 291 Wuhua Yellow chickens, yielding 11.16 million high-quality autosomal SNPs after quality control (mean depth 10.29×; Table S1). Principal component analysis (PCA) showed limited genome-wide stratification between single-comb and triple-comb individuals (Figure S1), and the first three principal components (PCs) were included as covariates in subsequent association analyses to control for population structure.

GWAS revealed a highly concentrated association signal on chromosome 19 (Figure 2A). A total of 8,588 SNPs exceeded the genome-wide significance threshold (*λ* = 0.921; Figure 2B), of which 8,585 (99.94%) mapped to a single 5.58-Mb interval (0.0276–5.607 Mb) (Table S2). Within this interval, the strongest associations clustered in a narrow core region overlapping *HSF5*, ENSGALG00000054410, and *MTMR4* (Figure 2C), and linkage disequilibrium (LD) analysis revealed an extended conserved haplotype surrounding the lead SNP (Figure 2D). A parallel GWAS using indel variants independently identified the same major locus on chromosome 19, with the strongest association signals concentrated in the same core region (Table S3).

**Figure 2:**
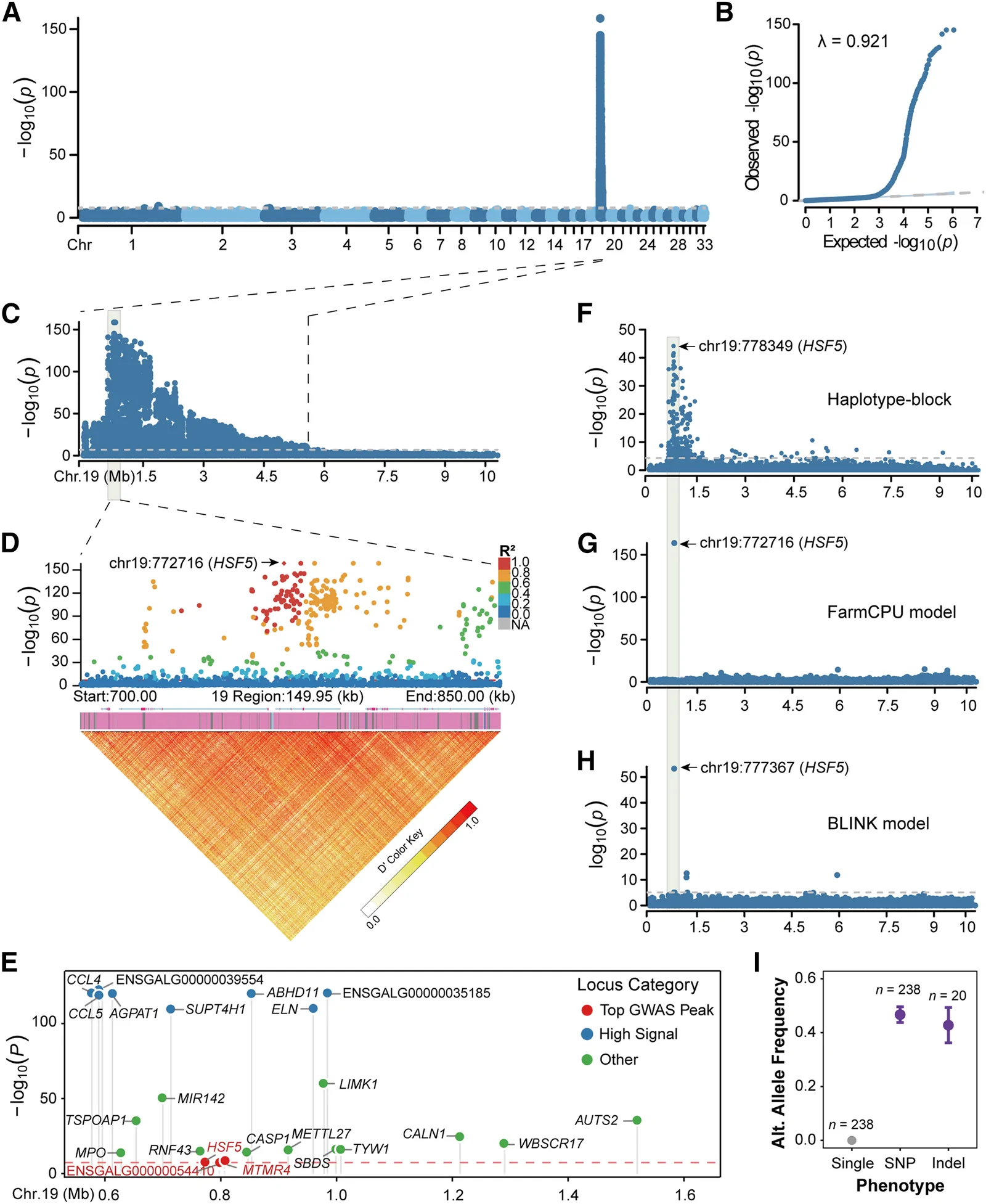
Genome-wide association study for the triple comb trait in chickens (A) Manhattan plot showing the genome-wide association results from the linear mixed model. Each point represents a SNP; its chromosomal position is on the x axis, and the association strength (−log_10_(*p* value)) is on the y axis. The gray horizontal line indicates the genome-wide significance threshold. (B) Quantile-quantile plot comparing the observed distribution of −log_10_(*p* values) (y axis) against the expected distribution under the null hypothesis (x axis). (C) Regional Manhattan plot focusing on the 5.58 Mb association interval on chromosome 19. (D) Linkage disequilibrium (LD) block structure (measured as *r*^2^) across the peak association region on chromosome 19. The plot is centered on the top-associated SNP (chr19: 772716, indicated by an arrow), revealing an extended, inverted-triangular region of high LD, which is indicative of a conserved, shared haplotype surrounding the candidate locus. The color gradient from red to white represents the strength of LD, ranging from high to low. (E) Lollipop plot from the conditional GWAS analysis. The plot displays the residual association strength (−log_10_(*p* value)) of each variant on the y axis against its genomic position on chromosome 19 (x axis) after conditioning on the top-associated SNP from the primary GWAS. (F–H) Haplotype-block association analysis for the chromosome 19 region performed using RAINBOWR. Association results for the chromosome 19 region from the FarmCPU and BLINK models implemented in GAPIT3 are shown in (G and H), respectively. (I) Alternative allele frequency (AF) for the top-associated SNPs (*n* = 238) and indels (*n* = 20) in triple-comb Wuhua Yellow chickens. These variants are fixed (AF = 0) in single-comb controls. Error bars represent ± one standard deviation (SD).

To dissect the genetic architecture of this locus, we performed conditional analyses using the most significant variant within each candidate gene as a covariate. For SNPs, the top variants in *HSF5*, ENSGALG00000054410, and *MTMR4* each reduced the core association signal below genome-wide significance (Figure 2E). However, for indels, only the top *HSF5* indel produced this effect (Figure S2), implicating *HSF5* as the gene harboring the causal variant.

Complementary haplotype-based and multi-locus models consistently identified *HSF5* as the top candidate, with association signals far exceeding those of other genes in the region (Figures 2F–2H; Table S4. Top haplotype blocks associated with comb morphology identified by genome-wide association study, related to Figure 2, Table S5. List of significant SNPs for chicken comb morphology identified by FarmCPU-GWAS on chromosome 19, related to Figure 2, Table S6. Significant loci identified by BLINK-GWAS for comb morphology on chromosome 19, related to Figure 2). Notably, analysis of the most significant GWAS SNPs and indels revealed that alternate alleles absent in single-comb individuals reached a frequency of only ∼0.47 in triple-comb birds (Figure 2I). This plateau at ∼0.5, rather than approaching fixation (∼1.0), indicates that no single SNP or indel fully accounts for the phenotype.

Taken together, these results strongly implicate the *HSF5* locus as the core candidate region. However, the findings also suggest that the underlying causal variants are not accounted for by simple SNPs or indels, prompting further investigation of SVs.

### SV analysis reveals a 15.7-kb tandem duplication upstream of *HSF5*

We examined the local sequence landscape for signatures of structural variation. Across the candidate region, triple-comb individuals showed marked distortion in allele-frequency patterns compared with single-comb birds, including depletion of intermediate-frequency variants within a narrow segment at approximately 0.78–0.80 Mb on chromosome 19 (Figures 3A and 3B). This pattern was accompanied by a discrete increase in local sequencing depth in triple-comb individuals, consistent with copy-number gain in the same interval (Figures 3C and 3D).

**Figure 3:**
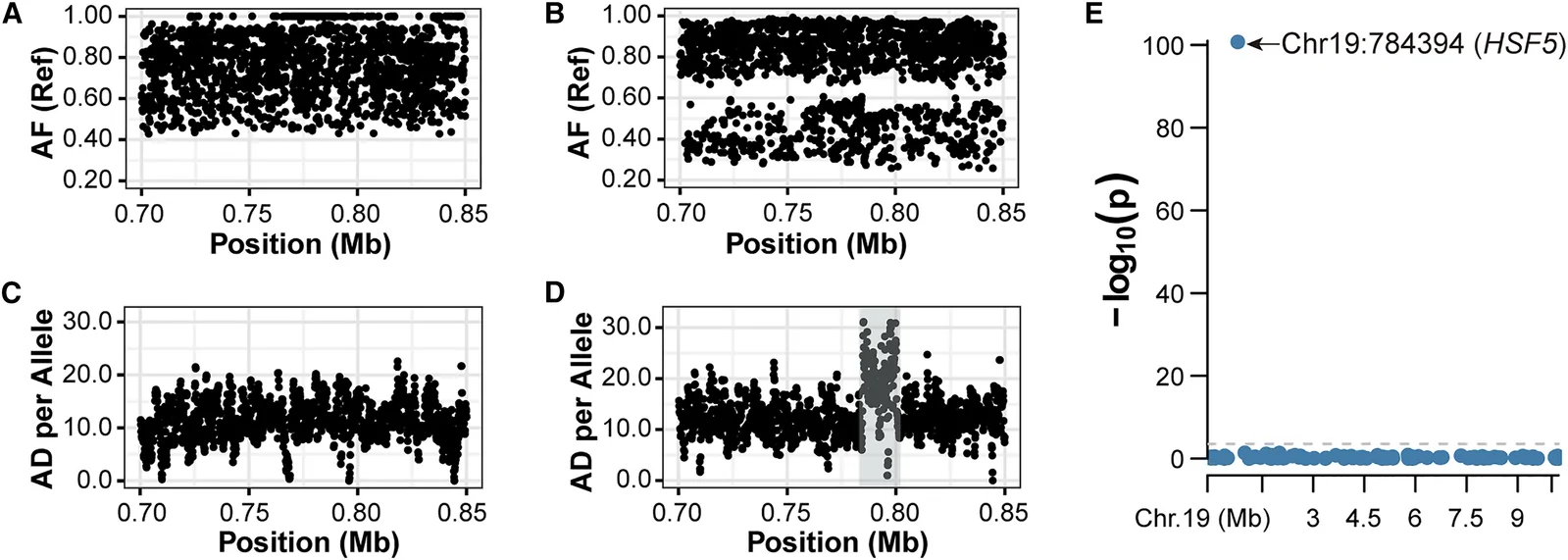
Identification of a tandem duplication at the *HSF5* locus (A) Distribution of reference allele frequency (AF) across the 0.70–0.85 Mb region on chromosome 19 in the single-comb population. (B) Distribution of reference AF across the same region in the triple-comb population. (C) Local average sequencing depth (AD) across the 0.70–0.85 Mb region in a representative single-comb individual. (D) Local AD across the same region in a representative triple-comb individual. (E) Genome-wide association results for structural variations on chromosome 19. Black arrow indicates the significantly associated 15.7-kb tandem duplication within the candidate region.

To test whether structural variation underlies the association signal, we performed targeted SV discovery on chromosome 19. This analysis identified 277 high-confidence structural variants, with only one showing remarkable association with the triple-comb phenotype: a 15.7-kb tandem duplication located upstream of *HSF5* (*p* = 1.81 × 10^−101^; Figure 3E). The duplication overlapped the core GWAS interval and represented the strongest candidate variant detected in this study.

In the discovery cohort, the tandem duplication was present in 62 of 66 triple-comb Wuhua Yellow chickens but absent in all 225 single-comb controls. The concordance among the SNP association peak, local copy-number signal, and SV association strongly implicates this 15.7-kb tandem duplication upstream of *HSF5* as the major candidate variant underlying the triple-comb phenotype.

### Long-read sequencing and population-scale genotyping validate the duplication

To resolve the structure of the candidate duplication and confirm its breakpoints, we performed PacBio high-fidelity (HiFi) sequencing on three triple-comb and two single-comb individuals (Table S7). Long-read alignments to the GRCg6a reference confirmed a tandem duplication spanning chr19: 784,394–800,058 in triple-comb individuals and its absence in single-comb controls (Figure 4A). Breakpoint-spanning reads established a head-to-tail arrangement of duplicated units and identified an 18-bp junction sequence connecting the two copies. Comparison with the reference genome revealed that the duplicated unit was 43 bp longer than the corresponding reference region due to several small insertions (Figure 4B; Table S8), providing base-pair-level resolution of the duplication architecture.

**Figure 4:**
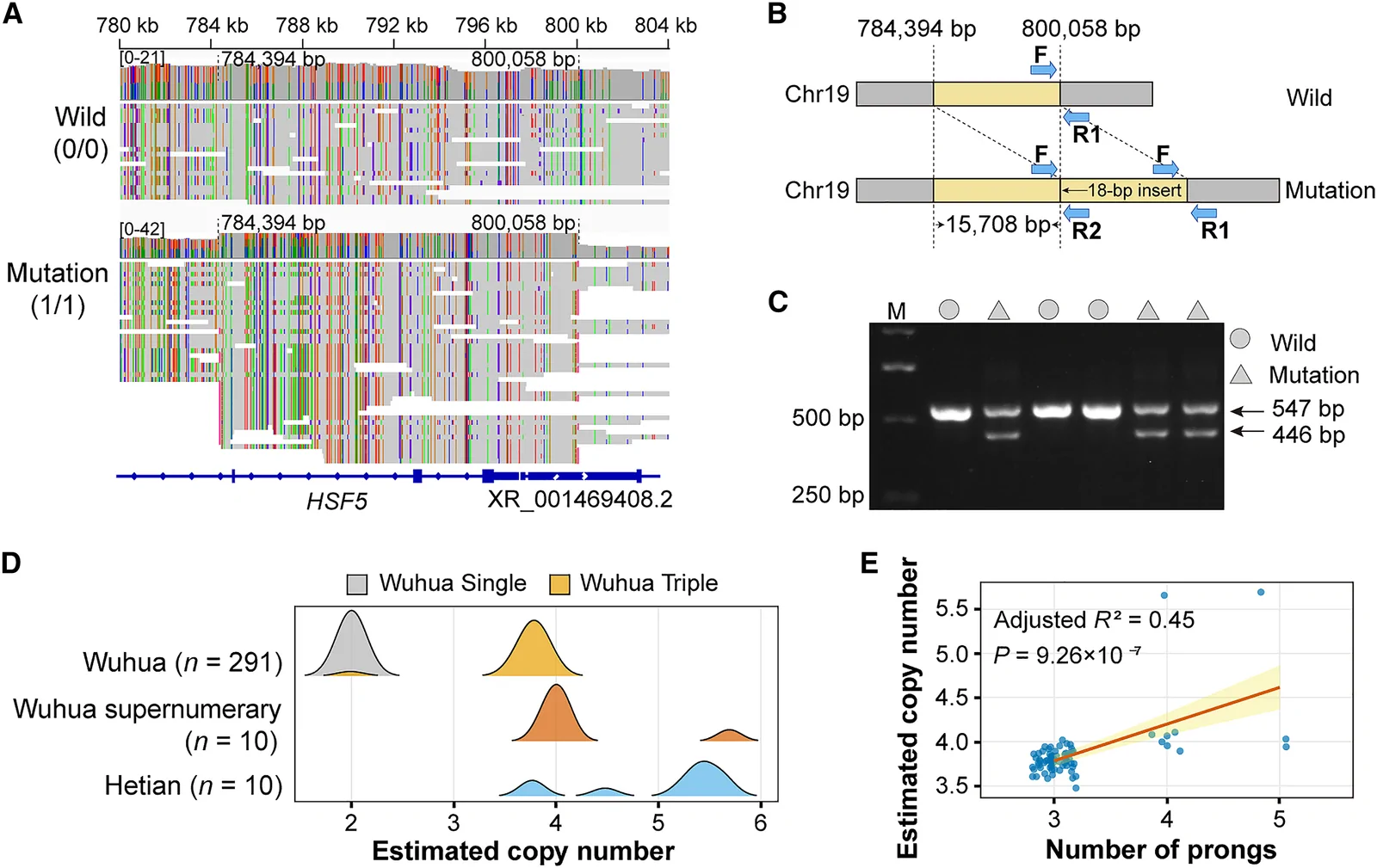
Structural validation, population genotyping, and dosage-dependent effect of the *HSF5* tandem duplication (A) Alignment of long-read HiFi sequencing data from single-comb (top) and triple-comb (bottom) individuals to the reference genome across the *HSF5* locus, confirming the tandem-duplication boundaries (chr19: 784,394–800,058) and organization. Gene annotations for *HSF5* and XR_001469408.2 are indicated. (B) Schematic of the tandem-duplication structure and PCR-genotyping strategy. Primer pairs are designed as follows: F-R1 amplifies a 547-bp fragment from both genotypes as an internal positive control, whereas F-R2 yields a 446-bp product only in the presence of the duplication. (C) Agarose gel electrophoresis of PCR products for genotyping the duplication. Lanes show the 547-bp control fragment (present in all samples) and the 446-bp duplication-specific fragment (present only in triple-comb individuals). M, DL 2,000 molecular marker. (D) Density distribution of estimated copy numbers for the *HSF5* duplication in single-comb (gray) and triple-comb (orange) Wuhua Yellow chickens (*n* = 291), Wuhua Yellow chickens with supernumerary prongs (*n* = 10, brown), and Hetian chickens (*n* = 10, blue). Dashed lines indicate copy numbers 2–6. (E) Correlation between estimated copy number and comb prong number in Wuhua Yellow chickens with the triple comb phenotype (prongs ≥3). Solid line represents the linear regression, and shaded area indicates the 95% confidence interval. Pearson’s correlation coefficient (*R*) and *p* value are shown.

We developed a junction-specific PCR assay for population-scale validation (Figure 4B; Table S9), amplifying a 547-bp internal control fragment in all individuals and a 446-bp duplication-specific fragment only in carriers. Consistent with long-read data, single-comb birds produced only the control amplicon, whereas duplication carriers produced both fragments (Figure 4C). Sanger sequencing of the duplication-specific product confirmed the predicted head-to-tail junction (Figure S3).

To assess the structural integrity at single-molecule resolution, we examined all HiFi reads spanning the *HSF5* locus. Junction-containing reads were detected in all three triple-comb samples (14/387, 10/346, and 9/355 reads, respectively) but not in single-comb controls. In two individuals (sample IDs: 0629 and 1930), reads containing two consecutive junction sequences (two reads each) were observed, indicating the presence of three tandem copies on at least one allele, whereas the third individual (sample ID: 2197) showed only single-junction reads.

We next evaluated the segregation of the duplication in expanded cohorts. In Wuhua Yellow chickens, the duplication-specific fragment was detected in 180 of 189 triple-comb individuals (95.2%) but in none of 1,367 single-comb controls (0%). In an independent population, all 75 tested Hetian chickens with bifurcated comb morphology carried the duplication (100%).

Thus, across a large number of samples and independent populations, the 15.7-kb tandem duplication showed a highly penetrant association with bifurcated comb phenotypes while remaining completely absent in the large single-comb Wuhua cohort. Together with SNP- and SV-based association evidence, these results highlight the duplication upstream of *HSF5* as the primary candidate variant underlying the triple-comb morphology.

### CNV at the *HSF5* duplication is associated with comb complexity

To further quantify CNV across populations, we performed read-depth-based CNV analysis in 291 Wuhua, 10 Wuhua Yellow chickens with supernumerary-prong combs, and 10 Hetian chickens, revealing distinct copy-number distributions across groups (Figure 4D). Single-comb individuals (*n* = 225) clustered at copy number 2, whereas most triple-comb Wuhua Yellow chickens (62/66) showed a shift to approximately 4 copies. The four PCR-negative triple-comb individuals clustered at a copy number of ∼2, indicating genetic heterogeneity within the phenotype. Given that the *CAMK2A* locus has previously been implicated in complex comb variations, we screened these four atypical individuals against 50 single-comb controls. Morphologically, these four duplication-negative birds met the standard criteria for fully developed bifurcated structures. Among 138 shared region-specific variants, only five were located within exons, all of which were benign synonymous, 3′ UTR, or intronic variants (Table S10). This analysis identified no shared high-impact coding or canonical splice-site variants within *CAMK2A*.

Wuhua Yellow chickens with supernumerary prongs (*n* = 10) were centered around a copy number of ∼4, with two individuals exhibiting higher values (∼5.7). Hetian chickens displayed a broader range of copy numbers (3.8–5.8).

Among duplication carriers spanning bifurcated and supernumerary-pronged morphologies, multiple linear regression adjusted for sex revealed that estimated *HSF5* copy number remained a highly significant independent predictor of comb prong number (*β* = 0.72, *p* = 9.26 × 10^−7^; adjusted *R*^2^ = 0.45; Figure 4E). Sex also exerted a significant effect, with males exhibiting higher prong numbers (*β* = 0.40, *p* = 4.14 × 10^−4^). This analysis was restricted to duplication carriers to specifically assess potential dosage effects. Complementing the regression analysis, copy numbers also differed across comb morphological categories (Kruskal-Wallis test, *p* = 0.014). Pairwise post hoc analyses were not performed due to limited sample sizes in higher-prong groups. Together, these findings provide supportive evidence for a dosage effect.

### Embryonic transcriptomic analyses reveal transcript remodeling of *HSF5* during comb development

To investigate the molecular consequences of the tandem duplication during comb development, we profiled gene expression in embryonic head crest tissue. Morphological examination showed that comb divergence became visually apparent from E12 onward (Figure 5A). Quantitative PCR using primers specifically targeting the duplicated segment (spanning ExonUnit6 and ExonUnit7) revealed that *HSF5* exhibited the strongest upregulation in triple-comb embryos across all stages, peaking at E8 (17.1-fold change, *p* < 0.01; Figure 5B). Neighboring genes showed relatively modest changes (Table S11; Figure 5C), prioritizing *HSF5* as the primary transcriptional target of the duplication.

**Figure 5:**
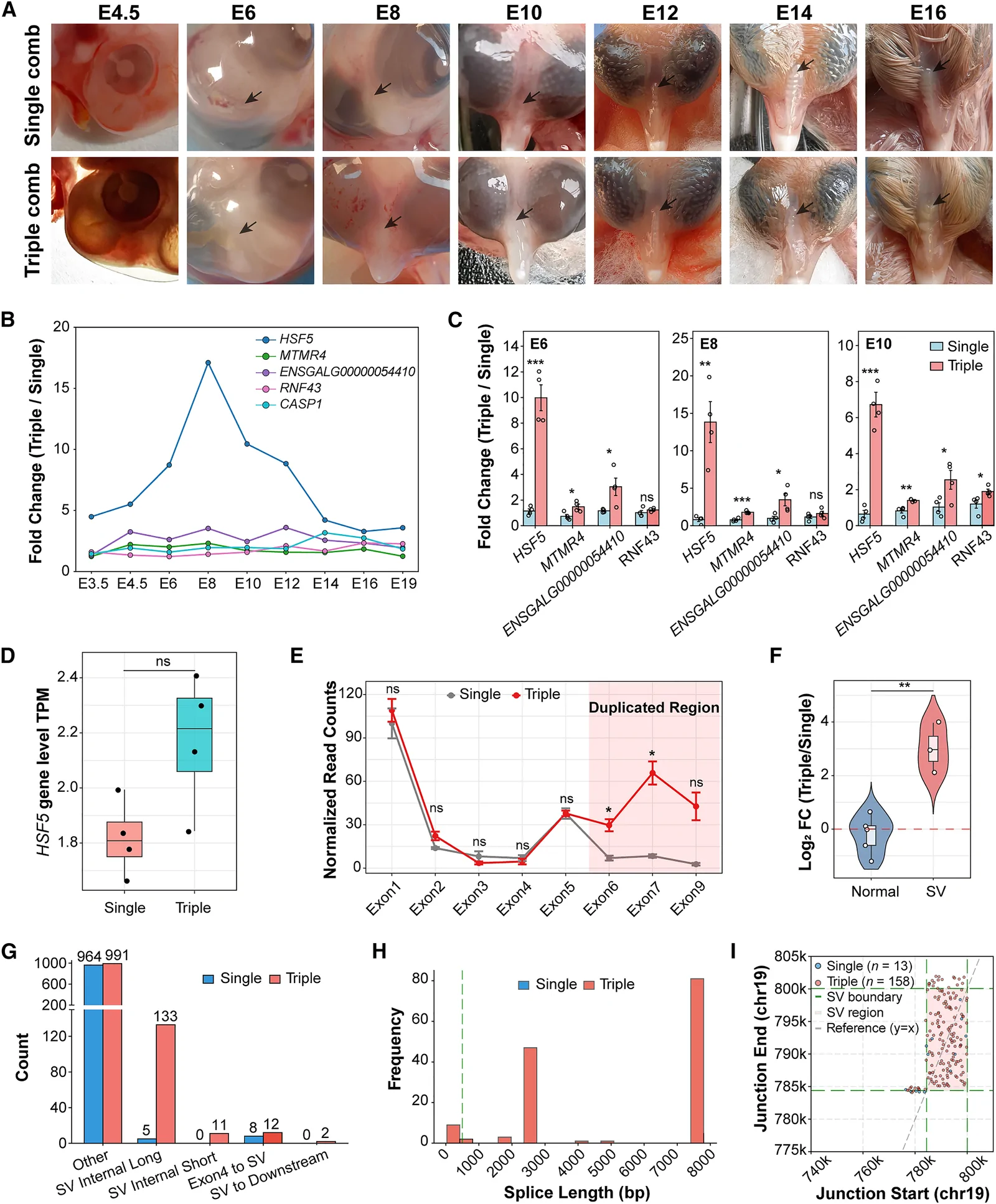
Developmental and molecular profiling of comb divergence (A) Morphological development of head crest tissues in single-comb and triple-comb embryos from E4.5 to E16. Phenotypic divergence becomes visually distinguishable from E12 onward. (B) Quantitative real-time PCR (qPCR) analysis of *HSF5* and four neighboring genes across developmental stages (E3.5–E19). Expression levels are shown as fold change of triple-comb relative to single-comb embryos for each stage. (C) Raw expression levels of *HSF5*, *MTMR4*, ENSGALG00000054410, and *RNF43* at the key stages E6, E8, and E10. Bar plots represent mean ± SEM; individual data points are shown as open circles. Statistical significance was determined by two-tailed Student’s *t* test (∗*p* < 0.05, ∗∗*p* < 0.01, ∗∗∗*p* < 0.001; ns, not significant). All qPCR assays were performed with four biological replicates per group per stage. (D) Whole-gene expression of *HSF5* at E8 by RNA sequencing (RNA-seq) transcripts per million (TPM). (E) Exon-level expression analysis of *HSF5* at E8. Trimmed mean of *M* values normalized read counts for each ExonUnit are shown for single-comb (gray) and triple-comb (red) embryos. (F) Comparison of log_2_(fold change, FC) between SV-region ExonUnits and non-SV ExonUnits. (G) Junction analysis showing SV-internal long splicing events. (H) Splice-length distribution of SV-internal junctions. (I) Junction positions plotted by genomic coordinates, with the SV region highlighted. Data are presented as mean ± SEM from *n* = 4 biological replicates per phenotype (or per stage per phenotype for qPCR), unless otherwise indicated. Statistical comparisons were performed using two-tailed Student’s *t* tests (∗*p* < 0.05, ∗∗*p* < 0.01, ∗∗∗*p* < 0.001; ns, not significant). For (G), junction-type enrichment was assessed by Fisher’s exact test with Bonferroni correction; for (E and F), *p* values were adjusted by the Benjamini-Hochberg method.

We then performed RNA sequencing (RNA-seq) on E8 head crest tissue (Table S12). Whole-gene expression of *HSF5* did not differ between phenotypes (Figure 5D). However, exon-level analysis, after TMM normalization based on exons outside the SV region (Table S13), revealed a spatially restricted pattern: ExonUnits within the duplicated region were elevated in triple-comb embryos, whereas those outside this interval showed no consistent difference (Figure 5E).

After multiple-testing correction, the expression of two ExonUnits within the duplicated region (ExonUnit6 and ExonUnit7) remained significantly elevated (adjusted *p* = 0.03; fold change: ExonUnit6 = 4.26×, ExonUnit7 = 7.84×) (Table S14). A third ExonUnit (ExonUnit9) showed a marked increase (fold change = 15.64×, nominal *p* = 0.02) but did not remain significant after correction (adjusted *p* = 0.07), likely due to low baseline expression. Allele-specific analysis confirmed an increasing expression gradient from ExonUnit6 to ExonUnit9 (4.17× → 6.91× → 7.38×) and a reversal of the ExonUnit6/ExonUnit5 ratio (0.57–1.98, *p* = 0.0038; Figure S4). Regional analysis showed significantly higher average log_2_(fold change) for SV-region ExonUnits than for non-SV ExonUnits (3.01 vs. −0.20, *p* = 0.0015; Figure 5F).

To further characterize transcript remodeling, we analyzed splice junction usage across the *HSF5* locus. Triple-comb embryos showed marked enrichment of non-canonical junctions within the duplicated interval, including a strong excess of long internal splice events (>500 bp) compared with single-comb embryos (133 vs. five events; 22.6-fold enrichment, *p* < 0.0001; Figure 5G). These novel junctions showed a broad distribution of splice lengths concentrated within the SV region (Figure 5H). Scatterplot analysis confirmed that these events were predominantly localized within the SV boundaries (chr19: 784,394–800,058), with minimal overlap in controls (Figure 5I).

To evaluate the potential protein-level consequences of these events, we performed *ab initio* gene prediction on the mutant genomic DNA using AUGUSTUS. This analysis predicted a functional open reading frame on the complementary strand encoding a potential 378-aa novel protein variant. Local sequence alignment with the wild-type HSF5 reference protein demonstrated complete sequence conservation across the core regions, confirming an intact, in-frame gene structure without premature termination. Furthermore, NCBI CD-Search verified that this predicted protein fully retains the highly conserved HSF DNA-binding domain (pfam00447; interval: 15–133 aa, *E* value = 2.67 × 10^−30^; Table S15), providing supportive *in silico* evidence for a gain-of-function model in which the structural variant expands the proteomic repertoire while preserving its primary DNA-binding capacity.

In summary, the tandem duplication leads to localized ExonUnit upregulation and is associated with novel non-canonical splicing patterns, and is predicted to generate a novel, structurally intact, in-frame *HSF5* protein variant during a critical window of comb morphogenesis.

### Epigenomic landscape of the 15.7-kb candidate region

To evaluate whether the 15.7-kb duplicated candidate interval harbors potential *cis*-regulatory elements, we examined its baseline epigenomic landscape using publicly available reference datasets. Chromatin state segmentation profiles revealed that the candidate region corresponds to active enhancer annotations across diverse chicken tissues (Figure 6A). Specifically, the region maps to predicted strong active enhancers (bright yellow) and medium active enhancers (brown), which co-localize with baseline signals of chromatin accessibility (ATAC-seq/DNase-seq) and active histone modifications (H3K27ac and H3K4me1). In addition, multi-tissue profiling from the ChickenGTEx database identified an H3K27ac enrichment peak centered within this window (chr19: 795,000–796,000; Figure 6B), which was present across 14 chicken tissues and showed notable signal intensity in the testis. Together, these public reference profiles suggest that the 15.7-kb candidate region encompasses an endogenous sequence with baseline active enhancer features under wild-type conditions, providing supportive context for its potential *cis*-regulatory role.

**Figure 6:**
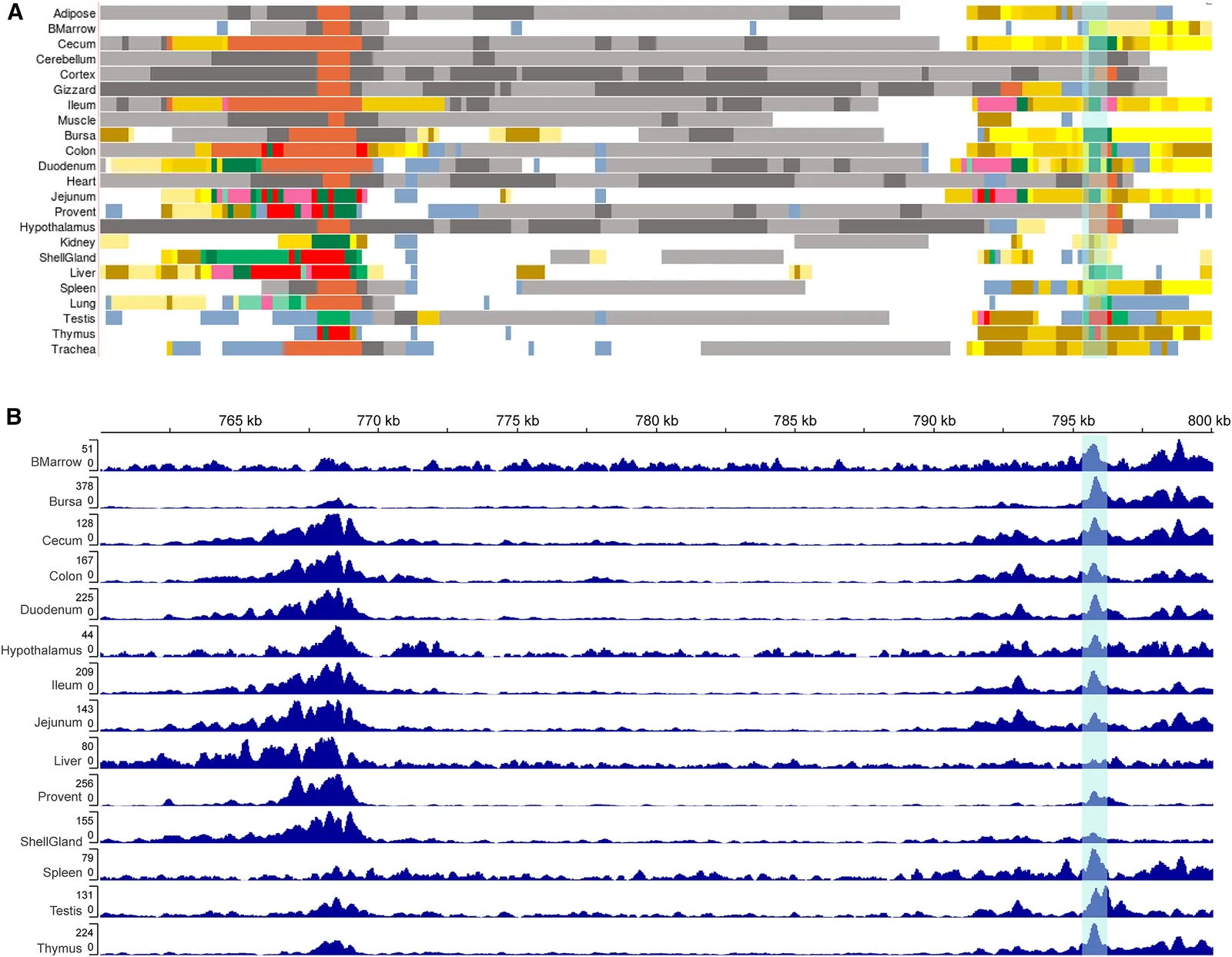
Baseline epigenomic characterization of the 15.7-kb candidate region upstream of *HSF5* in chickens (A) Multi-tissue chromatin state profiles across 23 chicken tissues. Colored segments represent predicted regulatory elements integrated by ChromHMM. Key functional categories include: active promoters (red/pink), active or primed enhancers (yellow/orange; yellow denotes strong active enhancers [EnhA], brown denotes medium enhancers [EnhAMe]), transcribed gene bodies (green), and quiescent or repressed regions (gray). The light-blue box highlights the 15.7-kb candidate region, which encompasses prominent active enhancer modules (yellow/orange) across multiple tissues. (B) Multi-tissue H3K27ac ChIP-seq signal profiles derived from ChickenGTEx across 14 chicken tissues. The light-blue box highlights a prominent, conserved active enhancer peak mapping directly within the 15.7-kb candidate interval.

### Population-genetic analyses support selection on the duplication-associated haplotype

To determine whether this regulatory duplication has been subject to selection during chicken breeding, we performed genome-wide selection scans across populations. Genome-wide population structure analysis confirmed the genetic distinctness of Wuhua Yellow chickens (PCA and ADMIXTURE; Figure S5), providing an appropriate framework for selection scans. Genome-wide *F*_ST_ analysis identified a prominent peak at the *HSF5* locus, and fine-scale mapping showed that this region coincided with the strongest genome-wide XP-CLR signal (Figures 7A and 7B; Tables S16 and S17). Analysis of Tajima’s D and π revealed elevated values in the triple-comb population relative to single-comb controls (Figures 7C and 7D; Tables S18 and S19). Although a classic hard sweep typically reduces genetic diversity, these elevated values are consistent with the complex genomic architecture of a tandem duplication, in which paralogous read alignment can artificially inflate regional diversity metrics.

**Figure 7:**
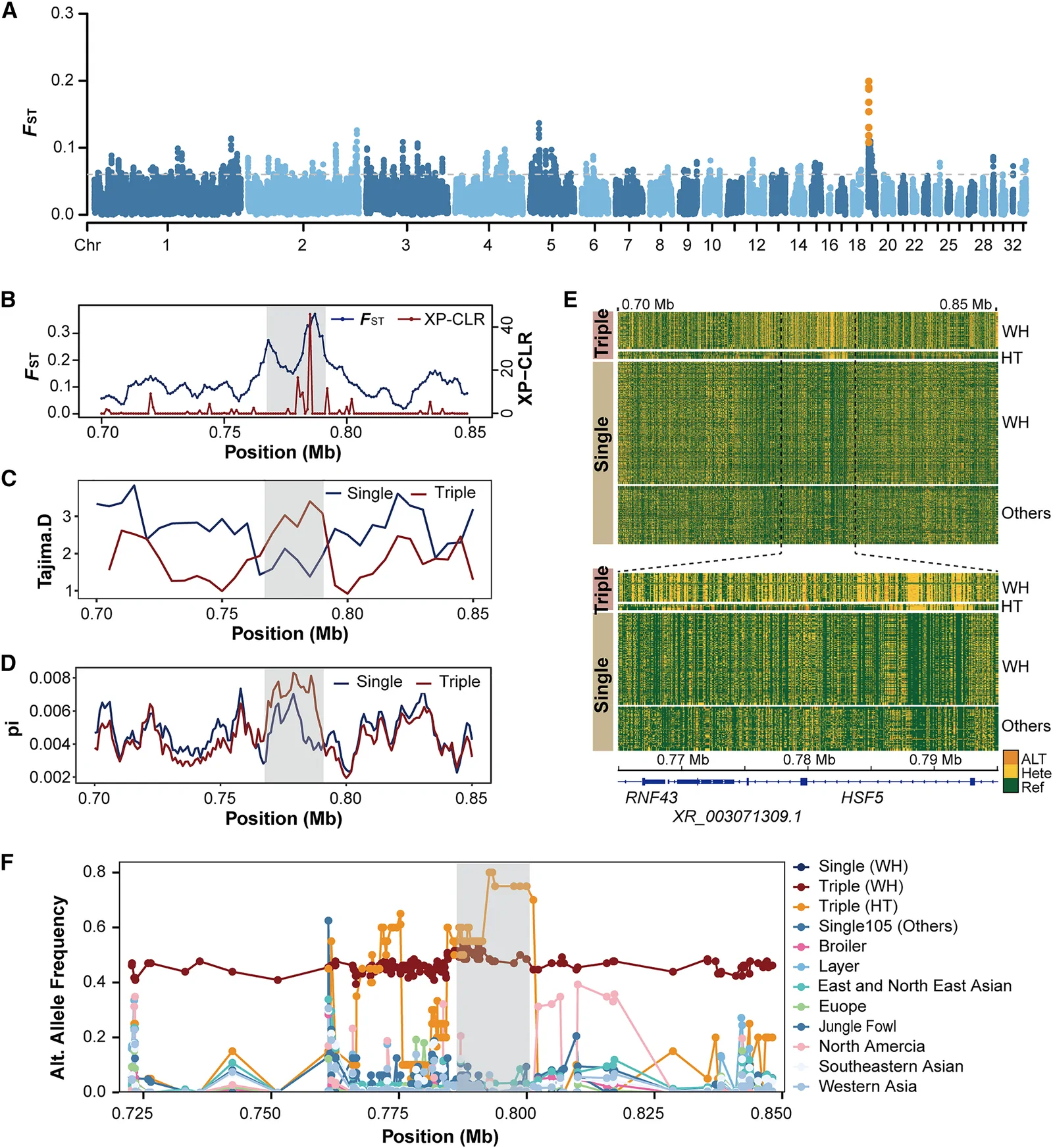
Population genetic signatures of selection at the *HSF5* locus (A) Genome-wide plot of genetic differentiation (*F*_ST_) between triple- and single-comb populations. The yellow highlight indicates the *HSF5* locus on chromosome 19, the primary candidate region identified in this study. (B) Fine-scale mapping of *F*_ST_ (dark blue line) and XP-CLR (dark red line) values across the candidate region on chromosome 19. (C and D) Sliding-window analysis of Tajima’s D and nucleotide diversity (π) across the *HSF5* locus for single-comb (dark blue) and triple-comb (dark red) populations. (E) Haplotype heatmap of the core selection region encompassing *HSF5* for the single- and triple-comb populations. (F) Allele frequencies of variants within the candidate region (chr19: 0.72–0.85 Mb) across multiple chicken populations. The core selective interval (0.78–0.80 Mb) is shaded in gray. Population abbreviations: WH, Wuhua Yellow chicken; HT, Hetian chicken.

Haplotype analysis revealed near-complete divergence between triple- and single-comb populations within the core region, with the triple-comb lineage dominated by a single haplotype (Figure 7E). Allele frequency spectra showed that trait-associated alleles were absent in single-comb Wuhua Yellow chickens but present at high frequency (∼0.47–0.53) in triple-comb individuals (Figure 7F). Hetian chickens exhibited comparable frequencies, consistent with a shared core haplotype across populations (Figure 7F).

Population screening confirmed the duplication in all 10 tested Hetian chickens with bifurcated comb morphology and its absence in all 24 single-comb breeds examined (Table S1). Analysis of 3,526 publicly available genomes from diverse global breeds detected the duplication in only 5 of 7 Hetian chickens, confirming its highly restricted distribution (Tables S20 and S21). Together with strong differentiation signals, these results support selective enrichment of the duplication-associated haplotype in lineages with bifurcated comb phenotypes.

### Exploratory fertility assessment in crosses involving the *HSF5* duplication

The strong selective signatures and prominent testis-specific activity of the duplication-associated haplotype led us to hypothesize a potential trade-off in male reproductive success. To test this hypothesis, we compared fertilization rates between triple- and single-comb crosses. In triple-comb crosses (154 eggs), 116 were fertilized (fertilization rate = 75.3%), of which 86 were confirmed by PCR genotyping to carry the duplication. The remaining 30 fertilized embryos could not be definitively classified owing to the presence of rare triple-comb individuals lacking the duplication. In single-comb crosses (108 eggs), 91 were fertilized (fertilization rate = 84.3%), and all were confirmed as wild-type at the *HSF5* locus. Fertilization rate was significantly lower in triple-comb crosses than in single-comb crosses (*χ*^2^ = 4.26, df = 1, *p* = 0.039). However, without direct measurements of specific sperm traits, these findings should be considered exploratory.

## Discussion

In this study, we resolved the genetic basis of triple-comb morphology in Wuhua Yellow chickens by integrating genome-wide mapping, structural variant discovery, long-read sequencing, population-scale validation, and embryonic transcriptomics. We identified a 15.7-kb tandem duplication upstream of *HSF5* on chromosome 19 as the major candidate variant associated with the trait, placing it within the established paradigm in which SVs underlie major chicken comb phenotypes through developmental regulatory perturbation.

### Confirmation of the associated locus and prioritization of *HSF5* as the primary effector

The 15.7-kb tandem duplication upstream of *HSF5* showed strong signatures of selection, including elevated *F*_ST_ and XP-CLR signals, along with near-complete haplotype divergence between phenotypes. The elevated π and Tajima’s D values in the triple-comb population, although atypical for a classic hard sweep, further support the complex architecture of tandem duplications.^23^^,^^24^^,^^25^^,^^26^ This pattern suggests that the locus underwent a complex process of genomic sorting or maintenance rather than a uniform selective sweep.

Several lines of evidence prioritized *HSF5* as the functional target. Fine-mapping localized the strongest signal immediately upstream of *HSF5*, and developmental expression profiling identified it as the gene with the most pronounced and temporally consistent expression differences. Critically, transcriptome profiling at E8 showed that only *HSF5* exhibited duplication-restricted exon upregulation and non-canonical splice junctions, representing a direct molecular signature of the SV. Neighboring genes displayed only modest and delayed upregulation consistent with downstream effects: *RNF43* peaked later (E10 vs. E8), *MTMR4* has no known role in appendage morphogenesis,^27^ and ENSGALG00000054410 remains uncharacterized. These patterns rule out the primary involvement of neighboring genes and identify *HSF5* as the principal effector, with secondary changes likely reflecting shared *cis*-regulatory perturbation.

The restricted distribution of the duplication further supported its functional relevance. Its high prevalence in lineages with bifurcated combs, absence in large single-comb cohorts, and rarity in global datasets (3,526 genomes) indicate lineage-specific enrichment under selection for ornamental traits. The discrepancy with the *CAMK2A* association reported in Qingyuan Partridge chickens^18^ likely reflects breed-specific genetic architecture,^11^^,^^28^ consistent with the presence of duplication-negative triple-comb individuals in our Wuhua cohort. Notably, screening these four atypical individuals against 50 single-comb controls revealed that, among 138 shared *CAMK2A* variants, only five were located within exons, all of which were benign synonymous, 3′ UTR, or intronic variants. Combined with the observation that these birds met the morphological criteria for fully bifurcated combs, these findings argue against direct structural disruption of the CAMK2A protein as the alternative driver. Instead, their phenotype may arise from cryptic structural variants in distant non-coding regulatory regions of *CAMK2A* or from independent, uncharacterized modifier loci that phenocopy the triple-comb morphology.

The *HSF5* duplication aligns the triple-comb phenotype with the established SV paradigm governing pea-, rose-, and duplex-comb phenotypes,^6^^,^^7^^,^^9^ while introducing a distinct mechanism. Unlike these variants, which are associated with ectopic gene expression, the *HSF5* duplication remodels transcript architecture, causing localized exon upregulation and novel splice variants, without altering overall gene expression. This expands the mechanistic scope of SV-driven phenotypic innovation, demonstrating that SVs can reshape genomic output at the transcript level. The integration of short- and long-read sequencing was essential to resolve this complex variant, as has been demonstrated for other traits such as fibromelanosis.^29^^,^^30^ Collectively, these findings strongly implicate *HSF5* as a key candidate gene within the SV-driven paradigm of comb diversity.

### Dosage-dependent phenotypic effect of the *HSF5* duplication

Duplication carriers exhibited elevated copy numbers across the interval, with a significant positive correlation between *HSF5* copy number and comb prong number. Consistently, the copy number also differed significantly across the comb morphological categories. This concordant pattern suggests that the tandem duplication may quantitatively contribute to comb complexity, supporting a model in which CNV is associated with the morphological continuum of bifurcated comb phenotypes.

The moderate strength of this relationship indicates that copy number is an important contributor but not the sole determinant of phenotypic outcome, consistent with the broad view that structural variants act within complex genetic and developmental contexts.^31^^,^^32^ A comparable example is observed in horses, where a tandem duplication in intron 6 of *Syntaxin 17* exhibits copy-number-dependent effects: two copies cause slow graying with low melanoma risk, whereas three copies lead to rapid graying and high melanoma incidence.^33^ Similarly, the fibromelanosis phenotype in chickens shows variable expression depending on genetic background.^34^ In this context, the *HSF5* duplication is likely associated with both the standard triple comb in Wuhua and the more elaborate deer-antler comb in Hetian, with phenotypic variation potentially reflecting breed-specific modifiers and/or developmental timing.

This potential dosage-dependent framework also helps interpret the variable expressivity observed in our data. The presence of a small number of triple-comb individuals lacking the duplication may reflect genetic heterogeneity or additional modifier loci influencing comb patterning, consistent with a complex genetic architecture. As in the horse model, where additional loci, such as *DPF3*, modulate melanoma risk,^35^ the incomplete correlation between *HSF5* copy number and comb complexity suggests the presence of unidentified modifiers.

### Molecular mechanisms of the causal variant and transcriptional remodeling

Our results further indicate that the tandem duplication is associated with transcript remodeling rather than a simple increase in whole-gene expression at the key embryonic stage examined. *HSF5* upregulation peaked at E8, a critical window for comb morphogenesis coinciding with feather tract patterning and dermal papilla formation.^36^ At this stage, total *HSF5* abundance did not differ significantly between phenotypes; however, ExonUnits within the duplicated region were consistently upregulated, with expression increasing progressively from ExonUnit6 to ExonUnit9. Concurrently, splice-junction analysis revealed extensive use of non-canonical junctions within the duplication, indicating the generation of novel isoforms. These patterns highlight two interconnected mechanisms: enhanced local transcription owing to increased enhancer dosage and SV-driven alternative splicing that expands the isoform repertoire.

Our characterization of public reference datasets further suggests that the 15.7-kb interval harbors endogenous *cis*-regulatory properties, as evidenced by active chromatin state annotations, high chromatin accessibility, and co-localized active histone modification signals (H3K27ac and H3K4me1). Although these baseline data reflect a non-variant genomic background, they demonstrate that the sequence itself exhibits active enhancer features under wild-type conditions. It is therefore plausible that tandem duplication of this interval increases the number of functional regulatory elements, potentially modulating local regulatory input and driving the observed transcript remodeling through an enhancer dosage effect, a regulatory phenomenon that has been well documented in other structural variant-mediated phenotypes.^31^^,^^37^^,^^38^ This localized transcript remodeling also explains the apparent discrepancy between our whole-genome RNA-seq and qPCR measurements, highlighting an important technical and biological distinction. Whereas whole-genome RNA-seq measures average read coverage across all 13 exons and therefore dilutes the signal from localized transcript changes, our qPCR assay specifically targeted ExonUnits 6–7 within the duplicated interval, thereby robustly capturing localized transcript enrichment.^39^

Given that tandem duplications are increasingly recognized as drivers of transcript diversity and functional innovation across species,^40^^,^^41^ duplication of this transcriptionally active sequence inherently expands the genomic substrate available for phenotypic innovation.^42^^,^^43^ Our *ab initio* gene prediction and domain analyses identified an intact, in-frame open reading frame that fully preserves the ancestral HSF DNA-binding domain. Mechanistically, the absence of premature termination codons allows this novel transcript to escape nonsense-mediated mRNA decay, potentially sustaining the observed regional upregulation. At the protein level, this structural framework supports a classic gain-of-function model. While the primary DNA-binding capacity is preserved, the elongated protein is predicted to introduce alternative macromolecular interaction surfaces. This structural extension may alter tertiary structural stability, protein half-life, or nucleocytoplasmic shuttling kinetics while facilitating non-canonical protein-protein interactions with somatic co-factors during critical embryonic windows.^41^^,^^42^ More broadly, these findings underscore how SV can reshape developmental output by topologically expanding, rather than disrupting, ancestral protein functions.^44^^,^^45^

*HSF5* encodes a heat shock transcription factor involved in chromatin remodeling and transcriptional reprogramming during meiosis.^46^^,^^47^^,^^48^^,^^49^ Although primarily characterized in germ cells, its regulatory capacity may influence developmental pathways when misexpressed in somatic tissues. In comb morphogenesis, altered *HSF5* activity may affect transcriptional programs governing epithelial-mesenchymal interactions. The Sonic hedgehog (SHH) pathway, implicated in pea-comb patterning through bone morphogenetic protein-SHH crosstalk and in duplex-comb formation,^8^^,^^9^ may represent a downstream convergence point. Dysregulation of HSF5 may alter chromatin states at loci involved in SHH or Wnt signaling, thereby reshaping morphogen gradients. Together, these findings posit *HSF5* within a regulatory framework in which SVs are associated with phenotypic innovation through transcript remodeling. This mechanism is distinct from the ectopic expression changes described for other comb variants, yet may converge on shared developmental pathways.

### Pleiotropic trade-offs and reproductive costs under artificial selection

Given the established role of *HSF5* in spermatogenesis across vertebrates,^47^^,^^48^^,^^49^ we assessed whether the duplication might affect reproductive traits. Fertilization rates were modestly but significantly lower in crosses involving triple-comb chickens than in single-comb controls, suggesting potential pleiotropic trade-offs. To provide a molecular context for this observation, we examined public reference datasets, which showed that *HSF5* expression and active H3K27ac signals are predominantly restricted to the testis.^50^ Although the lack of genotype-specific data in these public resources precludes definitive conclusions, this tissue-specific pattern supports the hypothesis that the structural duplication may disrupt normal spermatogenesis, providing a preliminary framework for the observed reduction in fertilization rates.^47^^,^^48^

In domestic chickens, major SVs underlying ornamental traits frequently have reproductive consequences. For example, the classic Rose-comb mutation, caused by a chromosomal inversion, leads to defective sperm motility and transient subfertility in homozygous roosters.^7^ The *HSF5* transcript remodeling identified here may represent a similar biological trade-off, in which intensive artificial selection for the ornamental triple-comb phenotype conflicts with optimal reproductive efficiency,^11^ providing a compelling example of the fitness costs associated with genetic hitchhiking under domestication. These findings, however, should be interpreted with caution, as direct assessments of sperm morphology, motility, or count were not performed. Crucially, embryos developed normally to the sampled stages and exhibited characteristic comb bifurcation from E12 onward, indicating that the primary impact of the duplication is on comb morphogenesis rather than embryonic viability.

In conclusion, we identified a 15.7-kb tandem duplication upstream of *HSF5* as the primary candidate variant underlying the triple-comb phenotype in chickens. This structural alteration is associated with transcript-architecture remodeling through alternative splicing and putative enhancer dosage effects, representing a mechanism distinct from classic sequence-based variation or simple ectopic gene expression. This study prioritizes *HSF5* as a key contributor to SV-driven comb morphologies and provides insights into the mechanisms through which structural variation and its associated regulatory contexts contribute to phenotypic diversity during domestication.

### Limitations of the study

This study has some limitations. First, the presence of duplication-negative triple-comb individuals suggests genetic heterogeneity. Second, causality requires functional validation, such as CRISPR-mediated deletion. Third, we did not distinguish direct *cis*-regulatory effects from secondary transcriptional cascades. Fourth, resolving the gene-dosage effect at the cellular level is constrained by early development; during the critical embryonic stages (E8–12), the comb primordium is too underdeveloped to predict future prong numbers, hindering targeted comparative histological analyses across continuous phenotypic gradients before these phenotypes fully manifest after hatching. Fifth, although fertilization rates were modestly reduced in triple-comb crosses, this analysis remains exploratory because male reproductive parameters (e.g., sperm characteristics) were not systematically quantified, leaving potential pleiotropic trade-offs unresolved. Sixth, the relatively small cohorts of local chicken breeds and the limited number of developmental qPCR replicates reduce statistical power and limit the generalizability of these findings to broader, genetically diverse populations.

Future studies should use *in vitro* cell models and spatial embryonic histology to dissect how variation in *HSF5* copy number modulates key cellular processes, such as cell proliferation and migration, during early development. Additionally, integrating CRISPR editing, reporter assays, and long-read isoform sequencing with expanded breed sampling (including both duplication-carrying and duplication-negative individuals) and crossbreed comparative genomics will improve statistical power and generalizability. Larger-cohort studies evaluating male reproductive traits are also needed to clarify potential breeding trade-offs under selection. From a practical perspective, the *HSF5* tandem duplication can serve as a precise molecular marker for marker-assisted selection, enabling early screening and potentially accelerating the breeding and fixation of the triple-comb trait. Together, these approaches will aid in elucidating the genetic architecture of bifurcated-comb phenotypes.

## Resource availability

### Lead contact

Requests for further information and resources should be directed to and will be fulfilled by the lead contact, Xun-He Huang (hxh826@jyu.edu.cn).

### Materials availability

This study did not generate new unique reagents.

### Data and code availability

• All sequencing data were deposited in the NCBI Sequence Read Archive under BioProject accession numbers PRJNA1433595, PRJNA1427308, and PRJNA1426986. The details of the aforementioned data used in this study are provided in Table S1.
• This paper does not report original code. All analyses were performed using publicly available software tools listed in the key resources table.
• Any additional information required to reanalyze the data reported in this paper is available from the lead contact upon request.

## Acknowledgments

This work was supported by the Peak Talent Program of Jiaying University (2022RC45), the Scientific Research Innovation Team Project of Jiaying University (2021JYUTDS04), and the Key Discipline Construction Project of Guangdong Provincial Department of Education (2022ZDJS088). We thank Luheng Zhang, Jiezhen Lin, and other colleagues for sample collection, Dr. Site Luo (Xiamen University) for bioinformatics support, and Run Wen for support in poultry management. We thank the Animal Bank at the Germplasm Bank of Wild Species (https://cstr.cn/31121.02.GBOWS.AB) for providing technical support.

## Author contributions

X.-H.H. and C.M. conceived and designed the project. X.-H.H., Z.-F.Z., Z.-P.Z., Z.-X.W., Y.-J.X., W.-N.L., and Q.W. collected experimental samples. X.-H.H., Z.-F.Z., Z.-P.Z., G.-H.Z., and C.M. performed data analysis. X.-H.H. and C.M. wrote the manuscript. X.-H.H., C.M., B.-W.D., and M.-S.P. participated in revising the manuscript.

## Declaration of interests

The authors declare no competing interests.

## Declaration of generative AI and AI-assisted technologies in the writing process

During the preparation of this work, the authors used Grok (xAI) to assist with language editing and improvement of readability. After using this tool, the authors carefully reviewed and edited the content as needed and take full responsibility for the content of the publication.

## STAR★Methods

### Key resources table

**Table:** 

REAGENT or RESOURCE	SOURCE	IDENTIFIER
**Biological samples**		

Wuhua Yellow chicken blood/embryo samples	Fengdu Breeding Farm, Meizhou, China	Tables S1, S7, and S12
Public chicken WGS data (24 breeds)	NCBI SRA	Table S1

**Chemicals, kits, and reagents**		

HiPure Universal DNA Kit	Guangzhou Magen Biotechnology Co., Ltd.	Cat# D3018
MGIEasy PCR-Free DNA Kit	BGI, Shenzhen	Cat# 1000013453
SMRTbell Express Template Prep Kit 2.0	PacBio	Cat# 100–950–300
TRIzol reagent	Invitrogen	Cat# 15596026
PrimeScript RT Reagent Kit	TaKaRa	Cat# RR037A
TB Green Premix Ex Taq II	TaKaRa	Cat# RR820A
DNase I	Invitrogen	Cat# AM2222

**Critical commercial assays**		

LightCycler 480 II qPCR system	Roche	N/A
DNBSEQ-T7 sequencing platform	BGI	N/A
PacBio Sequel II system	PacBio	N/A
Illumina NovaSeq 6000	Illumina	N/A

**Deposited data**		

Raw and processed sequencing data	This study	NCBI SRA: PRJNA1433595, PRJNA1427308, and PRJNA1426986
Public chicken WGS data	NCBI SRA	Table S1
Chicken reference genome GRCg6a	Ensembl	GCA_000002315.5

**Software and algorithms**		

fastp	https://github.com/OpenGene/fastp	v0.23.4
BWA-MEM	https://github.com/lh3/bwa	v0.7.17
Picard	https://broadinstitute.github.io/picard	v2.20.2
Genome Analysis Toolkit (GATK)	https://gatk.broadinstitute.org	v4.2.0.0
BCFtools	https://samtools.github.io/bcftools	v1.21
PLINK	https://www.cog-genomics.org/plink/	v1.90
GEMMA	https://github.com/genetics-statistics/GEMMA	v0.98.5
ANNOVAR	https://annovar.openbioinformatics.org	v20240219
LDBlockShow	https://github.com/hewm2008/LDBlockShow	v1.41
RAINBOWR	https://cran.r-project.org/package=RAINBOWR	v0.1.38
GAPIT	https://github.com/jiabowang/GAPIT	v3.5
smoove	https://github.com/brentp/smoove	v0.2.8
Delly	https://github.com/dellytools/delly	v1.7.2
minimap2	https://github.com/lh3/minimap2	v2.30
SAMtools	https://github.com/samtools/samtools	v1.21
cuteSV	https://github.com/tjiangHIT/cuteSV	v2.1.2
HISAT2	https://daehwankimlab.github.io/hisat2/	v2.2.1
featureCounts	https://subread.sourceforge.net	v2.0.3
edgeR	https://bioconductor.org/packages/edgeR	v3.40.2
bedtools	https://github.com/arq5x/bedtools2	v2.31.1
VCFtools	https://vcftools.github.io	v0.1.16
ADMIXTURE	https://dalexander.github.io/admixture	v1.3.0
XP-CLR	https://github.com/hardingnj/xpclr	v1.1.2
IGV	https://igv.org	v2.19.8
Ensembl Variant Effect Predictor	https://github.com/Ensembl/ensembl-vep	v110
CMplot	https://cran.r-project.org/package=CMplot	v4.5.1

**Other**		

Custom trio-primer PCR primers	This study	Table S9
Custom qPCR primers	This study	Table S9
Junction sequence for duplication detection	This study	TGGGTGAAAATGCAACTGTAATTTG GGTGAAAATGCAACTG

### Experimental model and study participant details

All animal experiments were approved by the Jiaying University Experimental Animal Ethics Committee (approval number: JYYXLL2025-13) and conducted in accordance with institutional regulations. Wuhua Yellow chickens were raised under uniform conditions in individual cages at the Fengdu Breeding Farm (Meizhou, China). At 20 weeks of age, mature comb morphology was assessed and classified as triple or single comb. Phenotypic classification and prong counting were performed consistently from a caudal-to-rostral view. Single-comb individuals were defined by a single, undivided posterior comb margin. Standard triple-comb individuals were characterized by a clear posterior bifurcation forming a three-ridged base. Supernumerary-prong variants were defined by the presence of additional secondary prongs branching independently from the lateral ridges or posterior margins, with the total number of distinct, elevated prongs recorded for each individual.

For GWAS, blood samples were collected from 66 triple-comb (13 males and 53 females) and 225 single-comb (225 females) Wuhua Yellow chickens. An additional 10 Wuhua Yellow chickens (seven males and three females) with supernumerary prongs (exhibiting four or more prongs according to the aforementioned counting criteria) were collected for copy-number correlation analysis. For population genetic analyses, we also retrieved whole-genome resequencing data of 105 single-comb chickens (representing 24 breeds) and 10 Hetian triple-comb chickens (10 females) from the NCBI Sequence Read Archive (Table S1). The integrated dataset comprised 231 individuals for downstream analysis, consisting of 76 triple-comb cases (66 Wuhua +10 Hetian) and 155 single-comb controls (50 Wuhua +105 public). For breeding experiments, crosses were established between triple-comb (eight roosters × five hens each) and single-comb (six roosters × five hens each) Wuhua Yellow chickens at 35 weeks of age; the single-comb breeders were obtained from the same farm but represent a separate cohort not included in the GWAS sequencing panel.

### Method details

#### Genotyping

Genomic DNA was extracted using a HiPure Universal DNA Kit (Guangzhou Magen Biotechnology Co., Ltd.) and assessed by agarose gel electrophoresis and a NanoDrop 2000 spectrophotometer. Libraries were prepared using an MGIEasy PCR-Free DNA Kit (BGI, Shenzhen, China) and sequenced on a DNBSEQ-T7 platform (PE150), achieving an average sequencing depth of 10× coverage per individual.

To broaden the genetic context for population and selection analyses, whole-genome resequencing data of 105 single-comb chickens representing 24 breeds and 10 Hetian chickens with triple-comb morphology were retrieved from the NCBI Sequence Read Archive (Table S1). The integrated dataset comprised 231 individuals: 76 triple-comb cases (66 Wuhua +10 Hetian) and 155 single-comb controls (50 Wuhua +105 public).

Raw reads were processed using fastp (v0.23.4) to remove low-quality sequences and adapters.^51^ The resulting clean reads were aligned to the chicken reference genome GRCg6a (GCA_000002315.5) using BWA-MEM (v0.7.17). Picard Tools (v2.20.2) was used to sort and merge alignment files and to remove PCR duplicates. Variant calling was performed using HaplotypeCaller (v4.2.0.0) in GATK.^52^ Variants were filtered using VariantFiltration in GATK with the following criteria: for single nucleotide polymorphisms (SNPs), “QD < 2.0 || MQ < 40.0 || FS > 60.0 || QUAL <30.0 || SOR >3.0 || MQRankSum < −12.5 || ReadPosRankSum < −8.0”; for insertion/deletion (indels), “QD < 2.0 || FS > 200.0 || SOR >10.0 || MQRankSum < −12.5 || ReadPosRankSum < −8.0”.

Quality control was performed using BCFtools (v1.21)^53^ to remove variants and samples with >10% missing data and retain only bi-allelic autosomal variants. For GWAS, we applied minor allele frequency (MAF >0.01) and Hardy–Weinberg equilibrium (*p* < 10^–6^) filters; for population genetic analyses, we used MAF >0.05 filtering to retain potential selection signals.

#### GWAS

GWAS for SNPs and indels was performed using a linear mixed model in GEMMA (v0.98.5),^54^ incorporating a kinship matrix and PCs as covariates. To determine the appropriate number of PCs for GWAS correction, we conducted PCA on 291 Wuhua Yellow chickens using PLINK (v1.90)^55^ with a pruned set of LD-independent SNPs (--indep-pairwise 50 10 0.1). The number of PCs was determined using the Tracy–Widom test (*p* < 0.05) and a scree plot. Genome-wide significance was defined by Bonferroni correction (*p* < 0.05/*N*, where *N* is the total number of tested variants). Results were visualized using the CMplot package (v4.5.1) in R. Significant variants were annotated using ANNOVAR (v20240219),^56^ and LD block structure was analyzed using LDBlockShow (v1.41).^57^

Conditional GWAS was performed by including the top variant as a fixed-effect covariate. Complementary analyses on chromosome 19 were conducted using haplotype-block GWAS in RAINBOWR (v0.1.38)^58^ as well as the FarmCPU and BLINK models implemented in GAPIT (v3.5)^59^ using the same Bonferroni threshold.

To evaluate alternative causal variants within the *CAMK2A* locus (chr13: 13,876,828–13,938,605; GRCg6a) in birds with atypical phenotypes, we performed targeted rare-variant filtration using BCFtools (v1.21).^53^ Genomic variants from the atypical individuals were jointly analyzed with a background control cohort of 50 normal single-comb chickens. Candidate variants were retained only if they were entirely absent from all 50 controls (homozygous wild type, 0/0) but present (heterozygous or homozygous) across all four atypical birds. Functional impacts of the filtered variants were predicted using the Ensembl Variant Effect Predictor (v110).^60^

#### Analysis of allele frequency spectrum

To evaluate the distribution and divergence of trait-associated alleles across diverse chicken populations and to fine-map regions of strongest phenotypic differentiation, we analyzed allele frequency spectra using a combination of in-house sequencing data and public reference panels. Allele frequencies for SNPs within the candidate region (chr19: 0.78–0.80 Mb) were derived from Wuhua case-control cohorts, selective sweep cohorts (including Hetian), and the Global Chicken Reference Panel.^61^ We calculated absolute allele-frequency differences (Δf) between single-comb Wuhua and each triple-comb group, prioritizing intervals with high Δf and low frequencies in global non-triple-comb breeds.

#### SV discovery, association, and validation

##### Identification and association testing of SVs

To investigate SVs as candidate causal mutations, we performed genome-wide SV discovery on chromosome 19 using smoove (v0.2.8), which implements LUMPY for SV calling,^62^ integrating split-read, read-pair, and read-depth signals to facilitate sensitive SV calling and genotyping from short-read sequencing data. We then conducted a focused GWAS for the triple-comb trait using SV genotypes in GEMMA, incorporating a kinship matrix and the first three PCs as covariates. The genome-wide significance threshold was determined using Bonferroni correction (*p* < 0.05/*N*, where *N* is the number of tested SVs).

##### Long-read sequencing for structural resolution

To resolve the precise boundaries and organization of candidate SVs, we performed long-read HiFi sequencing on three triple-comb (three males) and two single-comb individuals (one male and one female) at 20 weeks of age (Table S7). Libraries were prepared using a SMRTbell Express Template Prep Kit 2.0 (with size selection targeting fragment >10 kb) and sequenced on a PacBio Sequel II platform. HiFi reads were aligned to GRCg6a using minimap2 (v2.30)^63^ with the -ax map-hifi option, sorted using SAMtools (v1.21),^53^ and visualized in the Integrated Genomics Viewer (v2.19.8)^64^ to confirm tandem organization and define exact boundaries. Independent SV calling was performed using cuteSV (v2.1.2)^65^ with parameters optimized for HiFi reads (--max_cluster_bias_INS 1000 --diff_ratio_merging_INS 0.9 --max_cluster_bias_DEL 1000 --diff_ratio_merging_DEL 0.5).

To further assess the duplication structure at single-molecule resolution, we extracted reads spanning the *HSF5* locus (chr19: 600,000–1,000,000) and identified those containing the junction sequence connecting two consecutive copies (TGGGTGAAAATGCAACTGTAATTTGGGTGAAAATGCAACTG). These reads were classified based on the number of junction occurrences (with one indicating two tandem copies, and two indicating three tandem copies).

##### Experimental validation by PCR

The predicted tandem duplication was validated using a custom trio-primer PCR assay (Table S9). We used a forward primer at the 3′ end of the duplicated unit, paired with a common reverse primer in the adjacent single-copy region (serving as an internal control) and a duplication-specific reverse primer designed to bind within the second copy. The PCR reaction (30 μL) contained approximately 50 ng DNA, 0.2 μM of each primer, and 15 μL 2× Premix Taq (TaKaRa). Cycling conditions were as follows: 94°C for 5 min; 35 cycles at 94°C for 30 s, 58°C for 30 s, and 72°C for 30 s; followed by 72°C for 10 min.

Large-scale genotyping was performed using 75 Hetian (triple-comb), 189 Wuhua (triple-comb), and 1,367 Wuhua (single-comb) chickens. PCR products were resolved by agarose gel electrophoresis and scored for the presence of a 446-bp duplication-specific band.

#### Analysis of copy number variant

To quantify the copy number of the *HSF5* duplication across populations, we performed read depth-based CNV analysis using GATK (v4.2.0).^52^ Chromosome 19 was divided into 1,000 bp intervals using PreprocessIntervals, and read counts were collected using CollectReadCounts. A panel of normals was constructed from 225 single-comb Wuhua Yellow chickens using CreateReadCountPanelOfNormals.

For each individual, denoised log_2_ copy ratios were obtained using DenoiseReadCounts (with GC bias correction enabled). The average log_2_ ratio across the duplication region (chr19: 784,394–800,058) was calculated, and the copy number was estimated as 2 × 2ˆ (mean log_2_ ratio).

To test for a gene-dosage effect among duplication carriers, multiple linear regression was performed in R using the lm() function, with comb prong number modeled as a function of estimated copy number and sex (*Y* = comb_num ∼ est_cn + Sex). The independent effects of the predictors (β) and the overall model fit (adjusted *R*^2^) were evaluated. Differences in copy number among different comb morphological categories were further assessed using the Kruskal–Wallis test.

#### Embryo collection and assessment of fertilization

Crosses were established between triple-comb (eight roosters × five hens each) and single-comb (six roosters × five hens each) Wuhua Yellow chickens at 35 weeks of age. Fertilized eggs were collected daily and incubated at 37.8°C under 65% humidity. At embryonic day (E) 6, eggs were candled to assess fertility; those showing no development were opened to confirm lack of fertilization. The fertilization rate was calculated as the percentage of fertile eggs for each cross type. Embryos were harvested at nine developmental stages (E3.5, E4.5, E6, E8, E10, E12, E14, E16, and E19; *n* = 6–8 per stage per cross). Head crest tissues were dissected, immediately frozen in liquid nitrogen, and stored at −80°C. Phenotypes were confirmed by PCR genotyping prior to RNA extraction. Four embryos per stage per phenotype were selected for quantitative real-time PCR (qPCR).

#### qPCR analysis

qPCR was performed using embryonic head crest tissues without prior sex determination (*n* = 4 per stage per phenotype) to analyze *HSF5* and four neighboring genes (*MTMR4*, ENSGALG00000054410, *RNF43*, and *CASP1*). Total RNA was extracted using TRIzol reagent (Invitrogen), treated with DNase I, and reverse-transcribed using a PrimeScript RT Reagent Kit (TaKaRa). Gene-specific primers were designed using NCBI Primer-BLAST to target exons within the SV region (for *HSF5*) or conserved regions outside the SV (for neighboring genes) (Table S9). To capture localized expression changes within the SV, the *HSF5* qPCR primers were specifically designed to target the junction between ExonUnit6 and ExonUnit7, which directly overlaps with the 15.7-kb duplicated interval. qPCR was performed in triplicate on a LightCycler 480 II system (Roche) using TB Green Premix Ex Taq II (TaKaRa). Cycling conditions were as follows: 95°C for 30 s; 40 cycles at 95°C for 5 s and 60°C for 30 s; followed by a final melting curve analysis.

Relative gene expression was calculated using the 2ˆ^−ΔΔCt^ method with *GAPDH* as the endogenous control. *GAPDH* stability was confirmed using geNorm^66^ and low Ct variation. For each stage, the average ΔCt of the single-comb group served as the calibrator. Statistical comparisons were performed using two-tailed Student’s *t*-tests (*p* < 0.05).

#### RNA-seq and analysis of exon expression

RNA-seq was performed using E8 head crest tissues without prior sex determination (*n* = 4 per phenotype). Libraries were prepared by poly(A) selection and sequenced on an Illumina NovaSeq 6000 (PE150). All samples were processed in a single batch to minimize batch effects. Reads were aligned to the GRCg6a reference genome using HISAT2 (v2.2.1)^67^ with the --dta and --rna-strandness RF options.

Gene-level expression was quantified as transcripts per million (TPM) and compared between groups using Student’s *t*-tests. For exon-level analysis, *HSF5* exons were extracted, merged into non-redundant units, and categorized by their location relative to the SV region. Low-expression exons (counts <5 in ≥50% of samples) were excluded. Read counts were obtained using featureCounts (v2.0.3)^68^ with the parameters -p --countReadPairs -t exon -g gene_id. Normalization was performed using the trimmed mean of M-values (TMM) in edgeR (v3.40.2),^69^ based on exons located outside the SV region. Differential expression was assessed using *t*-tests with false discovery rate (FDR) correction where applicable.

Genotype-stratified regional expression was analyzed using bedtools coverage v.2.31.1^70^ and normalized to counts per million (CPM). Fold changes were calculated between triple- and single-comb embryos. Per-sample coverage values were extracted and visualized using boxplots to illustrate individual variation. To account for transcript heterogeneity, non-redundant “ExonUnits” were defined by intersecting the genomic coordinates of all four major annotated *HSF5* isoforms (Ensembl GRCg6a v110). An ExonUnit was defined as the maximum overlapping exonic interval shared among these isoforms (Table S13). Two ExonUnits flanking the structural variant (SV) were analyzed: ExonUnit5 (chr19: 779,423–779,968) and ExonUnit6 (chr19: 779,968–784,952). The ExonUnit6/ExonUnit5 ratio was calculated to control for global expression variation. Statistical significance was assessed using *t*-tests with Benjamini–Hochberg adjustment.

Split-read junctions were analyzed to identify alternative splicing. Junctions were extracted from BAM files across the *HSF5* locus and categorized into five types relative to SV boundaries: (i) long internal SV splicing (>500 bp), (ii) short internal SV splicing (≤500 bp), (iii) connections from ExonUnit5 (chr19: 779,423–779,968) to the SV region, (iv) connections from the SV region to downstream exons, and (v) canonical ExonUnit–ExonUnit6 splicing (chr19: 779,968–784,952). Enrichment was assessed using Fisher’s exact test with Bonferroni correction (*p* < 0.05/*N*, where *N* is the number of tested junction types). To ensure reproducibility, novel splice junctions were filtered using stringent criteria: a junction was considered valid only if it was supported by ≥ 3 unique split reads per sample and consistently detected in at least three of four biological replicates within the same phenotype group.

To characterize the potential translational products of these novel junctions, *ab initio* gene prediction was performed on the mutant genomic sequence using AUGUSTUS v.3.5.0 with chicken-specific parameters.^71^ Predicted open reading frames (ORFs) were evaluated for coding integrity and the presence of premature termination codons. The functional domain architecture of the predicted protein variant was subsequently analyzed using NCBI CD-Search against the Conserved Domain Database (v3.21) with an E-value threshold of 0.01.^72^

#### Population genetic and selective sweep analyses

To assess genome-wide population structure and detect signatures of selection, PCA and ADMIXTURE analyses were performed using the multi-breed dataset (231 samples). PCA was conducted using PLINK (v.1.90)^55^ on LD-pruned SNPs, as described above. Ancestry proportions were estimated using ADMIXTURE (v1.3.0)^73^ for *K* = 2–10, with the optimal *K* value determined by minimizing cross-validation error.

Selective sweep signals were quantified using multiple complementary statistics with optimized window parameters. Genetic differentiation (*F*_ST_) was calculated using VCFtools (v0.1.16)^74^ with a 50 kb sliding window and 10 kb step, a size commonly used in chicken genomics to obtain stable allele frequency estimates. Nucleotide diversity (π) was calculated using a 5 kb window and 1 kb step for fine-scale resolution. Tajima’s D was calculated using 10 kb windows as a complementary measure of selection. Cross-population composite likelihood ratio (XP-CLR) analysis was performed using a 10 kb window with a 5 kb step, following parameters optimized for detecting selective sweeps of moderate age in chicken populations.^75^

Genomic windows in the top 1% (or bottom 1% for Tajima’s D and π) of the genome-wide distribution were defined as outliers and considered candidate regions. Genes overlapping these regions were annotated using bedtools (v2.31.1)^70^ and the *Gallus gallus* GRCg6a genome annotation (Ensembl release 110).

#### Population-scale genotyping and prevalence analysis

The population specificity and prevalence of the tandem duplication were evaluated through bioinformatic genotyping of additional datasets, including selective sweep cohorts and 3,526 publicly available samples from diverse global breeds (Tables S18 and S19). SV calling within the target region (chr19: 600,000–1,000,000) was performed using smoove v.0.2.8 and Delly (v1.7.2).^76^ A sample was classified as carrying the duplication if at least one caller detected an event overlapping chr19: 784,394–800,058.

#### Public epigenomic data analysis and visualization

To evaluate the chromatin state and three-dimensional architecture of the *HSF5* locus, publicly available epigenomic datasets were integrated and analyzed. Chromatin accessibility (DNase-seq/ATAC-seq), histone modification profiles (H3K27ac, H3K4me1, H3K4me3, and H3K27me3), and CTCF-binding data across multiple chicken tissues were retrieved from the Chicken Regulatory Element Atlas.^77^ To further assess tissue-specific regulatory activity, multi-tissue H3K27ac ChIP-seq profiles and baseline *HSF5* mRNA expression across 14 distinct chicken tissues (including reproductive, immune, and metabolic organs) were obtained from the ChickenGTEx database (https://chicken.farmgtex.org/).^50^ All epigenomic tracks were visualized and aligned using the UCSC Genome Browser (https://genome.ucsc.edu/s/zhouhuaijun/chicken_regulatory_element).

### Quantification and statistical analysis

All statistical details, including the specific tests used, exact sample sizes (*n*), precision measures, and significance thresholds, are reported in the corresponding figure legends and Method Details. Statistical analyses were performed in R (v.4.2.2) unless otherwise noted. Parametric data were analyzed using two-tailed Student’s *t*-tests or multiple linear regression; non-parametric data were analyzed using Kruskal–Wallis tests; categorical data were evaluated using Fisher’s exact tests. Multiple testing corrections (Benjamini–Hochberg false discovery rate or Bonferroni) were applied where appropriate. Biological replicates are defined as independent biological samples collected from distinct individuals; technical replicates, when performed (e.g., qPCR), were averaged prior to statistical analysis.

## Supplemental information

Document S1. Figures S1–S6

Table S1. Summary of chicken genome sequencing samples used in this study, Related to STAR Methods and Figure 2

Table S2. Annotation of genome-wide suggestive or significant SNPs associated with comb traits, related to Figure 2

Table S3. Annotation of genome-wide suggestive or significant indels associated with comb traits, related to Figure 2

Table S4. Top haplotype blocks associated with comb morphology identified by genome-wide association study, related to Figure 2

Table S5. List of significant SNPs for chicken comb morphology identified by FarmCPU-GWAS on chromosome 19, related to Figure 2

Table S6. Significant loci identified by BLINK-GWAS for comb morphology on chromosome 19, related to Figure 2

Table S7. Summary of long-read HiFi sequencing data, related to Figure 4 and STAR Methods

Table S8. Sequence variants between the HSF5 duplication consensus and the reference genome, related to Figure 4

Table S9. Primer sequences used in this study, related to STAR Methods, Figure 4, and Figure 5

Table S10. Functional annotation of shared exonic variants within the CAMK2A locus across four atypical individuals, related to Figure 4

Table S11. Summary of qPCR expression statistics for HSF5 and neighboring genes across embryonic stages, related to Figure 5

Table S12. Summary of RNA-seq data for E8 head crest samples, related to Figure 5 and STAR Methods

Table S13. RNA-seq expression matrix of HSF5 ExonUnits in E8 head crest tissue, related to Figure 5

Table S14. Differential exon usage analysis of HSF5 at E8, related to Figure 5

Table S15. Amino acid sequences of the predicted protein isoforms derived from the mutant HSF5 locus, related to Figure 5

Table S16. Gene annotation of the top 1% FST regions on chromosome 19 associated with comb morphology, related to STAR Methods

Table S17. Top 1% genomic regions on chromosome 19 identified by XP-CLR selective sweep analysis between forked- and single-comb populations, related to STAR Methods

Table S18. Bottom 1% genomic windows with extreme π ratios between comb and single comb populations on chromosome 19, related to STAR Methods

Table S19. Genomic windows in the bottom 1% of Tajima’s D distribution on chromosome 19 between comb-type populations, related to STAR Methods

Table S20. Publicly available chicken genome sequencing samples used for structural variant detection, related to STAR Methods

Table S21. Sample information for SV detection from 863 genomes, related to STAR Methods

## References

[1] Johnsson M., Rubin C.-., Höglund A., Sahlqvist A.-., Jonsson K.B., Kerje S., Ekwall O., Kämpe O., Andersson L., Jensen P. (2014). The role of pleiotropy and linkage in genes affecting a sexual ornament and bone allocation in the chicken. Mol. Ecol..

[2] Al-Ramamneh D.S., Makagon M.M., Hester P.Y. (2016). The ability of White Leghorn hens with trimmed comb and wattles to thermoregulate. Poult. Sci..

[3] Qi K., Amevor F.K., Liu Z., He J., Xu D., Zhai C., Wang Y., Wu L., Wang Y., Shu G. (2025). Relationship between comb development, immune regulation, growth hormone, testosterone, and growth traits in Tianfu broilers. Poult. Sci..

[4] Bateson W. (1902). Reports to the Evolution Committee of the Royal Society*,* Report I.

[5] Bateson W., Punnett R.C. (1905). Reports to the Evolution Committee of the Royal Society, Report II.

[6] Wright D., Boije H., Meadows J.R.S., Bed'Hom B., Gourichon D., Vieaud A., Tixier-Boichard M., Rubin C.J., Imsland F., Hallböök F. (2009). Copy number variation in intron 1 of *SOX5* causes the pea-comb phenotype in chickens. PLoS Genet..

[7] Imsland F., Feng C., Boije H., Bed'Hom B., Fillon V., Dorshorst B., Rubin C.J., Liu R., Gao Y., Gu X. (2012). The *Rose-comb* mutation in chickens constitutes a structural rearrangement causing both altered comb morphology and defective sperm motility. PLoS Genet..

[8] Boije H., Harun-Or-Rashid M., Lee Y.J., Imsland F., Bruneau N., Vieaud A., Gourichon D., Tixier-Boichard M., Bed’hom B., Andersson L. (2012). Sonic hedgehog-signalling patterns the developing chicken comb as revealed by exploration of the *Pea-comb* mutation. PLoS One.

[9] Dorshorst B., Harun-Or-Rashid M., Bagherpoor A.J., Rubin C.J., Ashwell C., Gourichon D., Tixier-Boichard M., Hallböök F., Andersson L. (2015). A genomic duplication is associated with ectopic eomesodermin expression in the embryonic chicken comb and two duplex-comb phenotypes. PLoS Genet..

[10] Headon D. (2015). Morphological mutations: lessons from the cockscomb. PLoS Genet..

[11] Andersson L., Purugganan M. (2022). Molecular genetic variation of animals and plants under domestication. Proc. Natl. Acad. Sci..

[12] National Commission for Animal Genetic Resources (2025).

[13] Pearl R., Pearl M.D. (1909). Data on variation in the comb of the domestic fow. Biometrika.

[14] Steup H.H. (1924).

[15] Taylor L.W. (1946). Multiplex combs. Poult. Sci..

[16] Wei J. (2020).

[17] Luo W. (2021).

[18] Cheng Y. (2025).

[19] Huang X., Otecko N.O., Peng M., Weng Z., Li W., Chen J., Zhong M., Zhong F., Jin S., Geng Z. (2020). Genome-wide genetic structure and selection signatures for color in 10 traditional chinese yellow-feathered chicken breeds. BMC Genom..

[20] Huang X., Jiang Y., Zheng N., Liu H., Weng Z., Wen J., Li J., Ma C. (2025). Research note: Pool sequencing nominates *MITF* as a key candidate gene for white-belted feathers in chickens. Poult. Sci..

[21] Huang X., Zhang L., Zhong Z., Weng Z., Xu Y., Wen J., Ma C., Du B. (2026). *PMEL* governs autosomal dominant inheritance of white-tail independent of yellow body plumage in chickens (*gallus gallus domesticus*). Poult. Sci..

[22] Huang X., Weng Z., Xu Y., Wen J., Yang Y., Ma C., Luo S., Du B. (2026). Association of a synonymous *PMEL* variant with localized melanin deposition in chicken tail feathers. Anim. Genet..

[23] Rogers R.L., Cridland J.M., Shao L., Hu T.T., Andolfatto P., Thornton K.R. (2015). Tandem duplications and the limits of natural selection in *Drosophila yakuba* and *Drosophila simulans*. PLoS One.

[24] Liu S., Gao Y., Canela-Xandri O., Wang S., Yu Y., Cai W., Li B., Xiang R., Chamberlain A.J., Pairo-Castineira E. (2022). A multi-tissue atlas of regulatory variants in cattle. Nat. Genet..

[25] Bhati M., Mapel X.M., Lloret-Villas A., Pausch H. (2023). Structural variants and short tandem repeats impact gene expression and splicing in bovine testis tissue. Genetics.

[26] Hämälä T., Moore C., Cowan L., Carlile M., Gopaulchan D., Brandrud M.K., Birkeland S., Loose M., Kolář F., Koch M.A. (2024). Impact of whole-genome duplications on structural variant evolution in *Cochlearia*. Nat. Commun..

[27] Derkaczew M., Martyniuk P., Hofman R., Rutkowski K., Osowski A., Wojtkiewicz J. (2023). The genetic background of abnormalities in metabolic pathways of phosphoinositides and their linkage with the myotubular myopathies, neurodegenerative disorders, and carcinogenesis. Biomolecules.

[28] Lawal R.A., Martin S.H., Vanmechelen K., Vereijken A., Silva P., Al-Atiyat R.M., Aljumaah R.S., Mwacharo J.M., Wu D.D., Zhang Y.P. (2020). The wild species genome ancestry of domestic chickens. BMC Biol..

[29] Dorshorst B., Molin A.M., Rubin C.J., Johansson A.M., Strömstedt L., Pham M.H., Chen C.F., Hallböök F., Ashwell C., Andersson L. (2011). A complex genomic rearrangement involving the *endothelin 3* locus causes dermal hyperpigmentation in the chicken. PLoS Genet..

[30] Zhang J., Nie C., Li X., Zhao X., Jia Y., Han J., Chen Y., Wang L., Lv X., Yang W. (2022). Comprehensive analysis of structural variants in chickens using pacbio sequencing. Front. Genet..

[31] Spielmann M., Lupiá Ez D.G., Mundlos S. (2018). Structural variation in the 3D genome. Nat. Rev. Genet..

[32] Scott A.J., Chiang C., Hall I.M. (2021). Structural variants are a major source of gene expression differences in humans and often affect multiple nearby genes. Genome Res..

[33] Rubin C.J., Hodge M., Naboulsi R., Beckman M., Bellone R.R., Kallenberg A., J’Usrey S., Ohmura H., Seki K., Furukawa R. (2024). An intronic copy number variation in *Syntaxin 17* determines speed of greying and melanoma incidence in Grey horses. Nat. Commun..

[34] Ma C., Andersson L. (2025). Population genomic analysis identifies the complex structural variation at the fibromelanosis (*FM*) locus in chicken. Sci. Rep..

[35] Druml T., Brem G., Horna M., Ricard A., Grilz-Seger G. (2022). DPF3, a putative candidate gene for melanoma etiopathogenesis in gray horses. J. Equine Vet. Sci..

[36] Hamburger V., Hamilton H.L. (1951). A series of normal stages in the development of the chick embryo. J. Morphol..

[37] Lupiáñez D., Kraft K., Heinrich V., Krawitz P., Brancati F., Klopocki E., Horn D., Kayserili H., Opitz J., Laxova R. (2015). Disruptions of topological chromatin domains cause pathogenic rewiring of gene-enhancer interactions. Cell.

[38] Siraj L., Castro R.I., Dewey H.B., Kales S., Butts J.C., Nguyen T.T.L., Kanai M., Berenzy D., Mouri K., Wang Q.S. (2026). Functional dissection of complex trait variants at single-nucleotide resolution. arXiv.

[39] Lio C.T., Düz T., Hoffmann M., Willruth L.L., Baumbach J., List M., Tsoy O. (2025). Comprehensive benchmark of differential transcript usage analysis for bulk and single-cell RNA sequencing. NAR Genom. Bioinform.

[40] Ivanov T.M., Pervouchine D.D. (2022). Tandem exon duplications expanding the alternative splicing repertoire. Acta Nat..

[41] Mantica F., Irimia M. (2025). Gene duplication and alternative splicing as evolutionary drivers of proteome specialization. Bioessays.

[42] Bush S.J., Chen L., Tovar-Corona J.M., Urrutia A.O. (2017). Alternative splicing and the evolution of phenotypic novelty. Phil. Trans. R. Soc. B..

[43] Martinez Gomez L., Pozo F., Walsh T.A., Abascal F., Tress M.L. (2021). The clinical importance of tandem exon duplication-derived substitutions. Nucleic Acids Res..

[44] Jumper J., Evans R., Pritzel A., Green T., Figurnov M., Ronneberger O., Tunyasuvunakool K., Bates R., Žídek A., Potapenko A. (2021). Highly accurate protein structure prediction with AlphaFold. Nature.

[45] Korchak J.A., Yi S.S., Kelleher N.L., Sahni N., Sheynkman G.M. (2026). Proteoform medicine: characterizing and targeting protein forms in human disease. Nat. Rev. Genet..

[46] Saju J.M., Hossain M.S., Liew W.C., Pradhan A., Thevasagayam N.M., Tan L.S.E., Anand A., Olsson P.E., Orbán L. (2018). Heat shock factor 5 is essential for spermatogenesis in zebrafish. Cell Rep..

[47] Luo C., Xu H., Yu Z., Liu D., Zhong D., Zhou S., Zhang B., Zhan J., Sun F. (2024). Meiotic chromatin-associated HSF5 is indispensable for pachynema progression and male fertility. Nucleic Acids Res..

[48] Yoshimura S., Shimada R., Kikuchi K., Kawagoe S., Abe H., Iisaka S., Fujimura S., Yasunaga K.i., Usuki S., Tani N. (2024). Atypical heat shock transcription factor HSF5 is critical for male meiotic prophase under non-stress conditions. Nat. Commun..

[49] Luo C., Fan Z., Yu Z., Liu D., Xu H., Zhou S., Zhu X., Liu H., Shao L., Li Z. (2025). Testis-specific protein HSF5 is essential for proper chromatin organization and transcriptional reprogramming to drive pachynema progression. Zool. Res..

[50] Hou Y., Zou D., Chu Q., Zhan B., Wang R., Guan D., Wang W., Feng X., Li X., Zhu X. (2026). The ChickenGTEx portal: a pan-tissue catalogue of regulatory variants shaping transcriptomic and phenotypic diversity. Nucleic Acids Res..

[51] Chen S. (2023). Ultrafast one-pass FASTQ data preprocessing, quality control, and deduplication using fastp. iMeta.

[52] Mckenna A., Hanna M., Banks E., Sivachenko A., Cibulskis K., Kernytsky A., Garimella K., Altshuler D., Gabriel S., Daly M. (2010). The Genome Analysis Toolkit: A MapReduce framework for analyzing next-generation DNA sequencing data. Genome Res..

[53] Danecek P., Bonfield J.K., Liddle J., Marshall J., Ohan V., Pollard M.O., Whitwham A., Keane T., Mccarthy S.A., Davies R.M. (2021). Twelve years of SAMtools and BCFtools. GigaScience.

[54] Zhou X., Stephens M. (2012). Genome-wide efficient mixed-model analysis for association studies. Nat. Genet..

[55] Purcell S., Neale B., Todd-Brown K., Thomas L., Ferreira M.A.R., Bender D., Maller J., Sklar P., de Bakker P.I.W., Daly M.J. (2007). PLINK: a tool set for whole-genome association and population-based linkage analyses. Am. J. Hum. Genet..

[56] Wang K., Li M., Hakonarson H. (2010). ANNOVAR: functional annotation of genetic variants from high-throughput sequencing data. Nucleic Acids Res..

[57] Dong S.S., He W.M., Ji J.J., Zhang C., Guo Y., Yang T.L. (2021). LDBlockShow: a fast and convenient tool for visualizing linkage disequilibrium and haplotype blocks based on variant call format files. Brief. Bioinform..

[58] Hamazaki K., Iwata H. (2020). RAINBOW: Haplotype-based genome-wide association study using a novel SNP-set method. PLoS Comput. Biol..

[59] Wang J., Zhang Z. (2021). GAPIT version 3: boosting power and accuracy for genomic association and prediction. Genom. Proteom. Bioinform..

[60] McLaren W., Gil L., Hunt S.E., Riat H.S., Ritchie G.R.S., Thormann A., Flicek P., Cunningham F. (2016). The ensembl variant effect predictor. Genome Biol..

[61] Zhu D., Wang Y., Qu H., Feng C., Zhang H., Sheng Z., Jiang Y., Nie Q., Chu S., Shu D. (2025). GCRP: integrated global chicken reference panel from 11,951 chicken genomes. Genom. Proteom. Bioinform..

[62] Layer R.M., Chiang C., Quinlan A.R., Hall I.M. (2014). LUMPY: a probabilistic framework for structural variant discovery. Genome Biol..

[63] Li H. (2018). Minimap2: pairwise alignment for nucleotide sequences. Bioinformatics.

[64] Thorvaldsdóttir H., Robinson J.T., Mesirov J.P. (2013). Integrative genomics viewer (IGV): high-performance genomics data visualization and exploration. Brief. Bioinform..

[65] Jiang T., Liu Y., Jiang Y., Li J., Gao Y., Cui Z., Liu Y., Liu B., Wang Y. (2020). Long-read-based human genomic structural variation detection with cuteSV. Genome Biol..

[66] Vandesompele J., De Preter K., Pattyn F., Poppe B., Van Roy N., De Paepe A., Speleman F. (2002). Accurate normalization of real-time quantitative RT-PCR data by geometric averaging of multiple internal control genes. Genome Biol..

[67] Pertea M., Kim D., Pertea G.M., Leek J.T., Salzberg S.L. (2016). Transcript-level expression analysis of RNA-seq experiments with HISAT, StringTie and Ballgown. Nat. Protoc..

[68] Liao Y., Smyth G.K., Shi W. (2014). featureCounts: an efficient general purpose program for assigning sequence reads to genomic features. Bioinformatics.

[69] Robinson M.D., Mccarthy D.J., Smyth G.K. (2010). edgeR: a bioconductor package for differential expression analysis of digital gene expression data. Bioinformatics.

[70] Quinlan A.R., Hall I.M. (2010). BEDTools: a flexible suite of utilities for comparing genomic features. Bioinformatics.

[71] Stanke M., Keller O., Gunduz I., Hayes A., Waack S., Morgenstern B. (2006). AUGUSTUS: *ab initio* prediction of alternative transcripts. Nucleic Acids Res..

[72] Wang J., Chitsaz F., Derbyshire M.K., Gonzales N.R., Gwadz M., Lu S., Marchler G.H., Song J.S., Thanki N., Yamashita R.A. (2023). The conserved domain database in 2023. Nucleic Acids Res..

[73] Alexander D.H., Novembre J., Lange K. (2009). Fast model-based estimation of ancestry in unrelated individuals. Genome Res..

[74] Danecek P., Auton A., Abecasis G., Albers C.A., Banks E., DePristo M.A., Handsaker R.E., Lunter G., Marth G.T., Sherry S.T. (2011). The variant call format and VCFtools. Bioinformatics.

[75] Chen H., Patterson N., Reich D. (2010). Population differentiation as a test for selective sweeps. Genome Res..

[76] Rausch T., Zichner T., Schlattl A., Stütz A.M., Benes V., Korbel J.O. (2012). DELLY: structural variant discovery by integrated paired-end and split-read analysis. Bioinformatics.

[77] Pan Z., Wang Y., Wang M., Wang Y., Zhu X., Gu S., Zhong C., An L., Shan M., Damas J. (2023). An atlas of regulatory elements in chicken: A resource for chicken genetics and genomics. Sci. Adv..

