# Supplementary material for: Tandem duplication upstream of HSF5 associates with transcript remodeling and triple-comb morphology in chickens
Source: iScience. 2026 Sep 28;29(10):117678. doi: 10.1016/j.isci.2026.117678 (PMC13639972; doi:10.1016/j.isci.2026.117678)
Supplement: Supplementary file 1 [file mmc1.pdf]

iScience, Volume *29*

Supplemental information

Tandem duplication upstream of *HSF5* associates with transcript remodeling and triple-comb morphology in chickens

Xun-He Huang (黄勋和), Zhi-Feng Zhang (张志锋), Zhi-Peng Zhong (钟志鹏), Zhuo-Xian Weng (翁茁先), Yong-Jie Xu (徐勇洁), Guo-Hao Zhong (钟国豪), Wei-Na Li (李威娜), Qing Wang (王庆), Bing-Wang Du (杜炳旺), Min-Sheng Peng (彭旻晟), and Cheng Ma (马成)

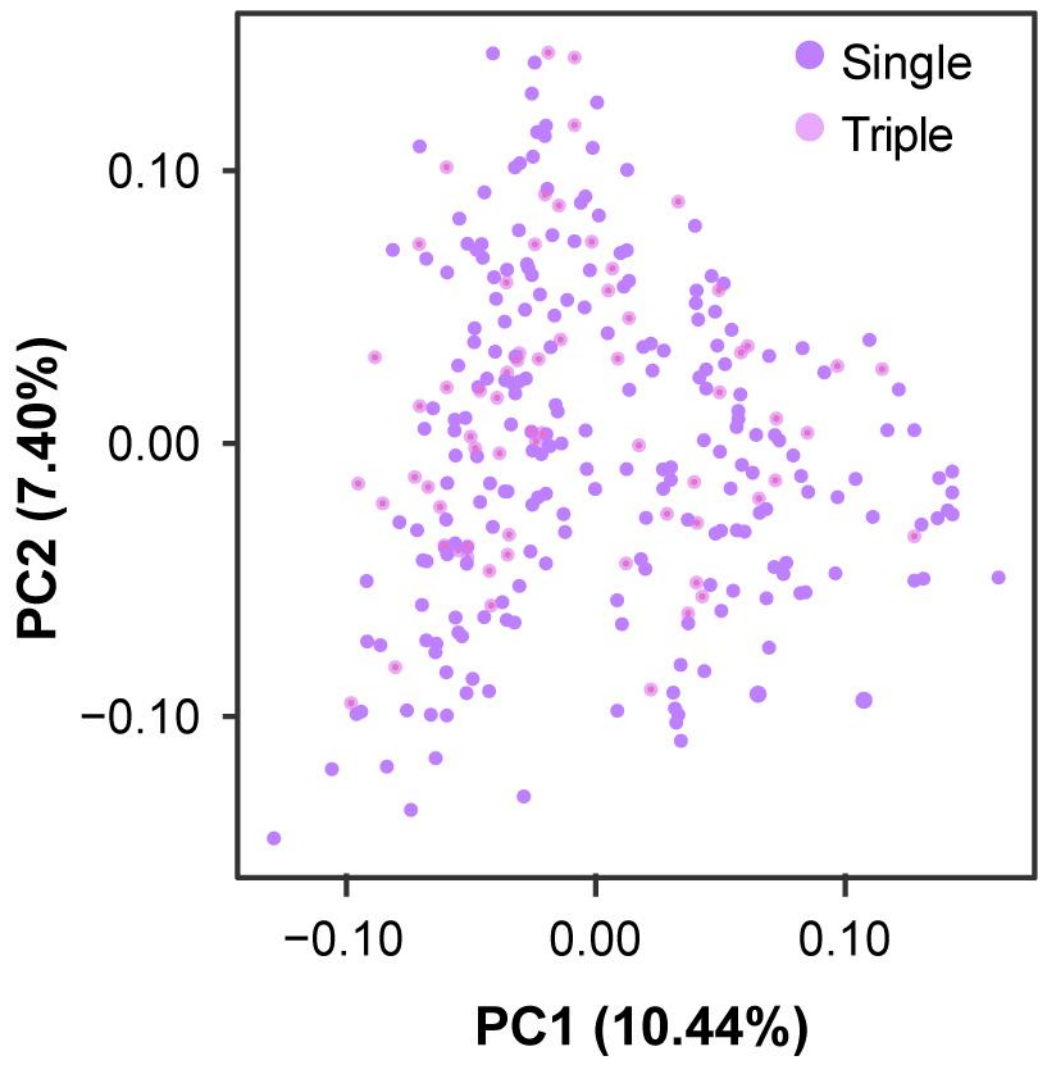

Figure S1. Principal component analysis of Wuhua Yellow chickens with single and triple combs based on genome-wide SNPs

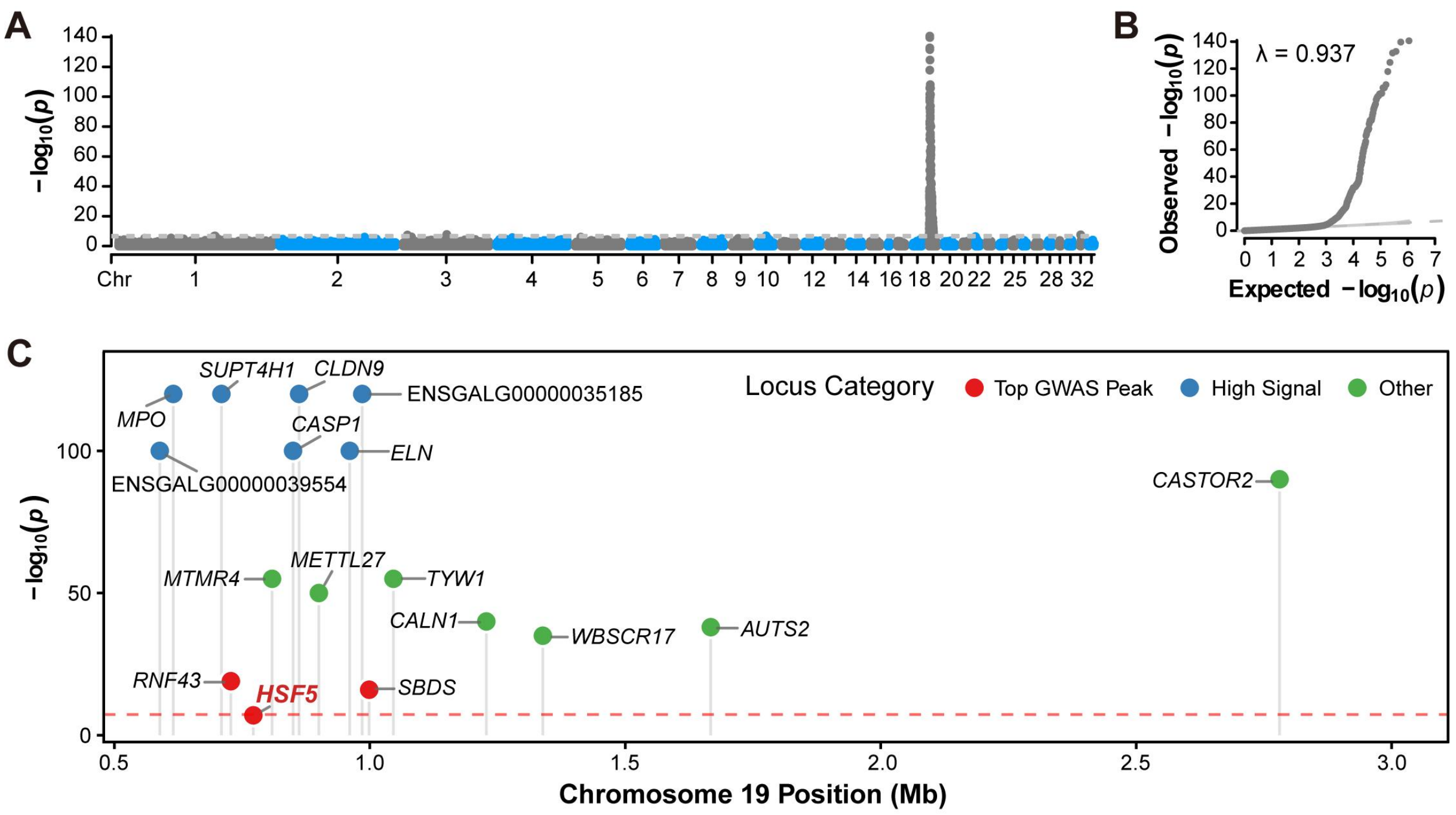

Figure S2. Genome-wide association study for the triple comb trait in chickens

(A) Manhattan plot showing the genome-wide association results from the linear mixed model. Each point represents an indel; its chromosomal position is on the x-axis, and the association strength (-$\log_{10}$(P-value)) is on the y-axis. The grey horizontal line indicates the genome-wide significance threshold. (B) Quantile-quantile plot comparing the observed distribution of -$\log_{10}$(*P*-values) (y-axis) against the expected distribution under the null hypothesis (x-axis). (C) Lollipop plot from the conditional GWAS analysis. The plot displays the residual association strength (-$\log_{10}$(*P*-value)) of each variant on the y-axis against its genomic position on chromosome 19 (x-axis) after conditioning on the top-associated indel from the primary GWAS.

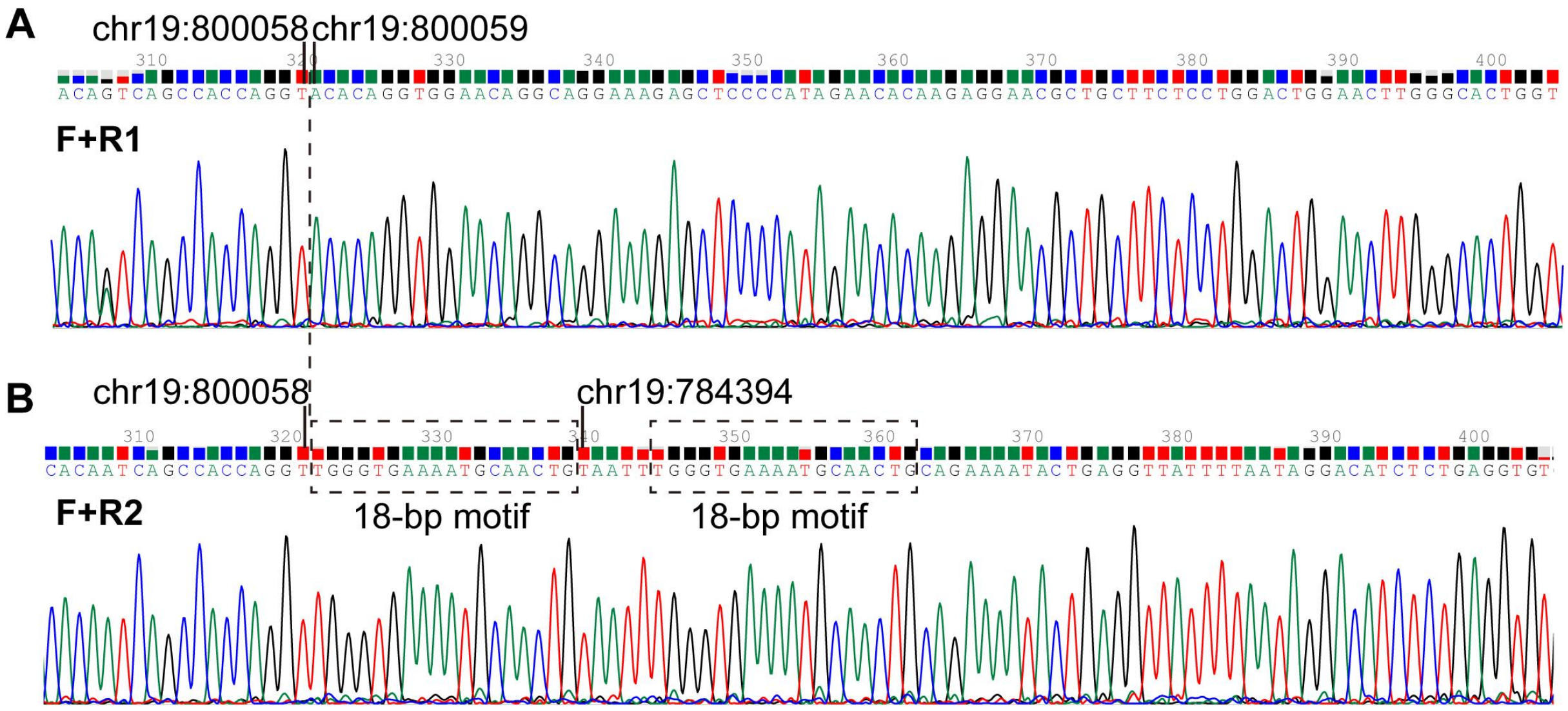

Figure S3. Sanger sequencing validation of the *HSF5* tandem duplication structure

(A) Control sequence spanning the normal genomic boundary at chr19:800,058/800,059 in single-comb individuals, showing the expected contiguous sequence of the wild-type allele without duplication. (B) Duplication-specific sequence from triple-comb individuals, revealing the tandem organization of the duplicated segment (chr19:784,394–800,058). The two copies of the duplicated unit are separated by an 18-bp spacer sequence (5′-TGGGTGAAAATGCAACTG-3′), indicated by the dashed box. The left breakpoint (chr19:800,058) and right breakpoint (chr19:784,394) are joined via this spacer to form the junction between the two duplicated copies, confirming the head-to-tail tandem arrangement of the duplication.

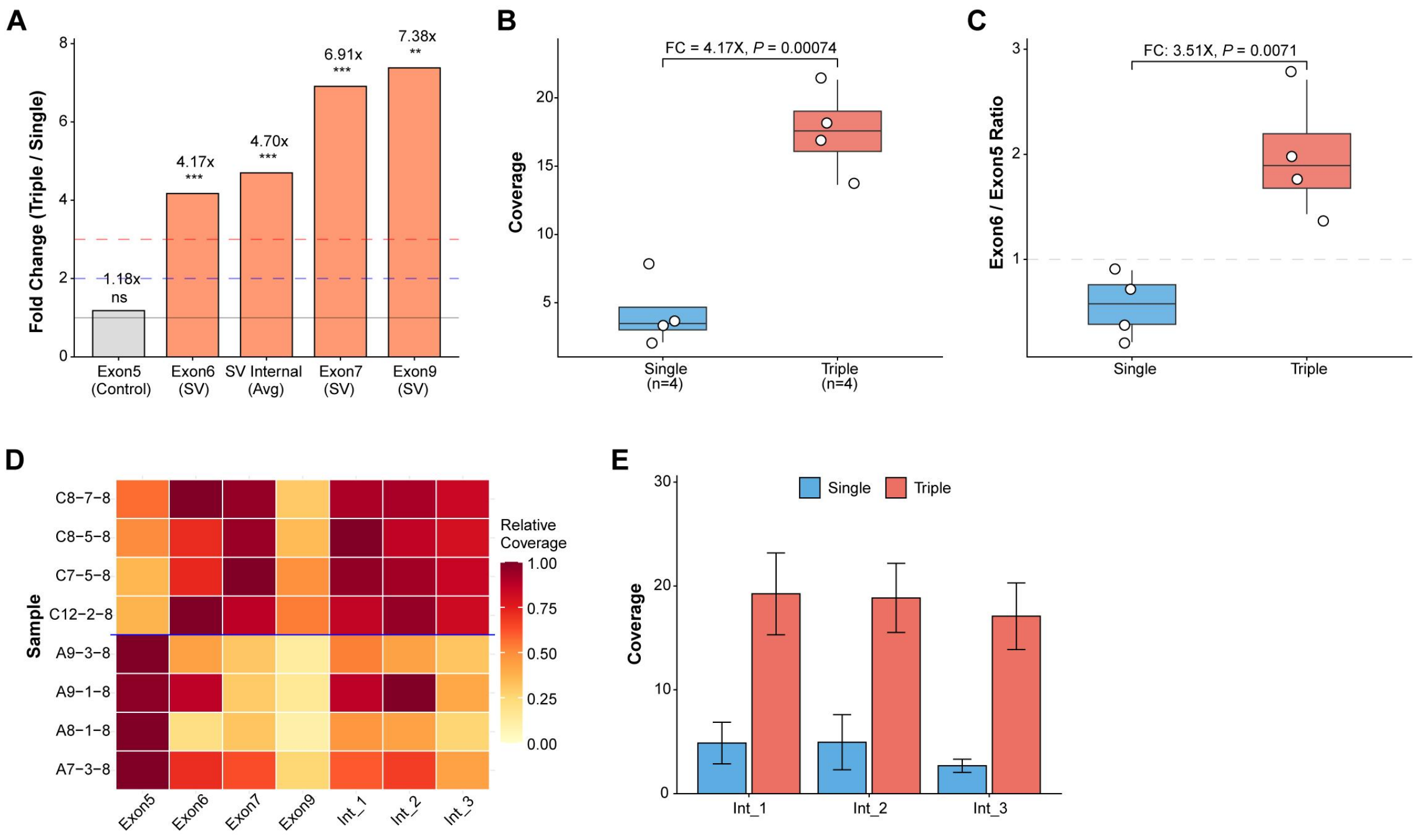

Figure S4. Allele-specific expression analysis reveals SV-driven mechanisms of *HSF5* overexpression

(A) Fold change in coverage for each genomic region (Triple/Single). Exon5 served as a control (1.18×, not significant). SV-associated regions showed significant upregulation: Exon6 (4.17×), Exon7 (6.91×), Exon9 (7.38×), and SV internal intervals (average 4.70×). Statistical significance was assessed by two-sample t-tests; ***$P < 0.001$, **$P < 0.01$. (B) Individual sample coverage values for SV_Exon6. Box plots show median and quartiles with individual samples labeled. Fold change = 4.17×, P = 0.0006. (C) Normalized Exon6 expression (Exon6/Exon5 ratio). The ratio reversed from <1 in single-comb to >1 in triple-comb embryos (fold change = 3.51×, P = 0.0038), indicating SV-driven 3' enrichment. (D) Heatmap of relative coverage across all samples and regions. The blue line separates single-comb (top) and triple-comb (bottom) samples. (E) SV internal intervals (Int_1-3) showing consistent upregulation in triple-comb samples, supporting active transcription throughout the duplicated region.

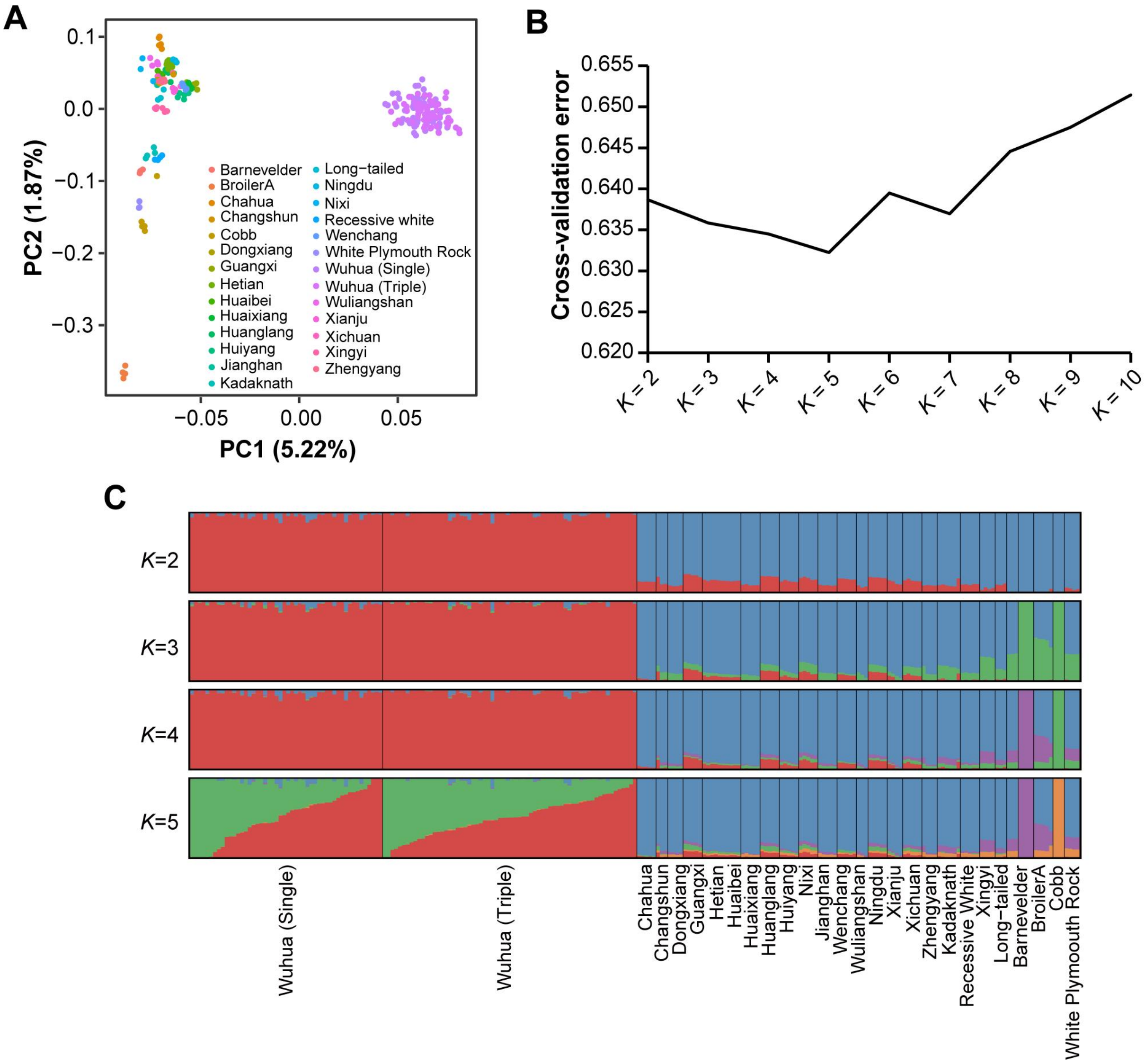

Figure S5. Population structure analysis of 231 multi-breed samples

(A) Principal component analysis of 231 samples from 26 breeds, with different colors representing distinct breeds. (B) Cross-validation error was calculated for each *K* value from 2 to 10. The lowest error was observed at *K* = 5, indicating the optimal number of ancestral clusters for the dataset of 231 individuals. (C) Ancestry proportions estimated by ADMIXTURE analysis across a range of ancestral populations (*K* = 2 to 5).

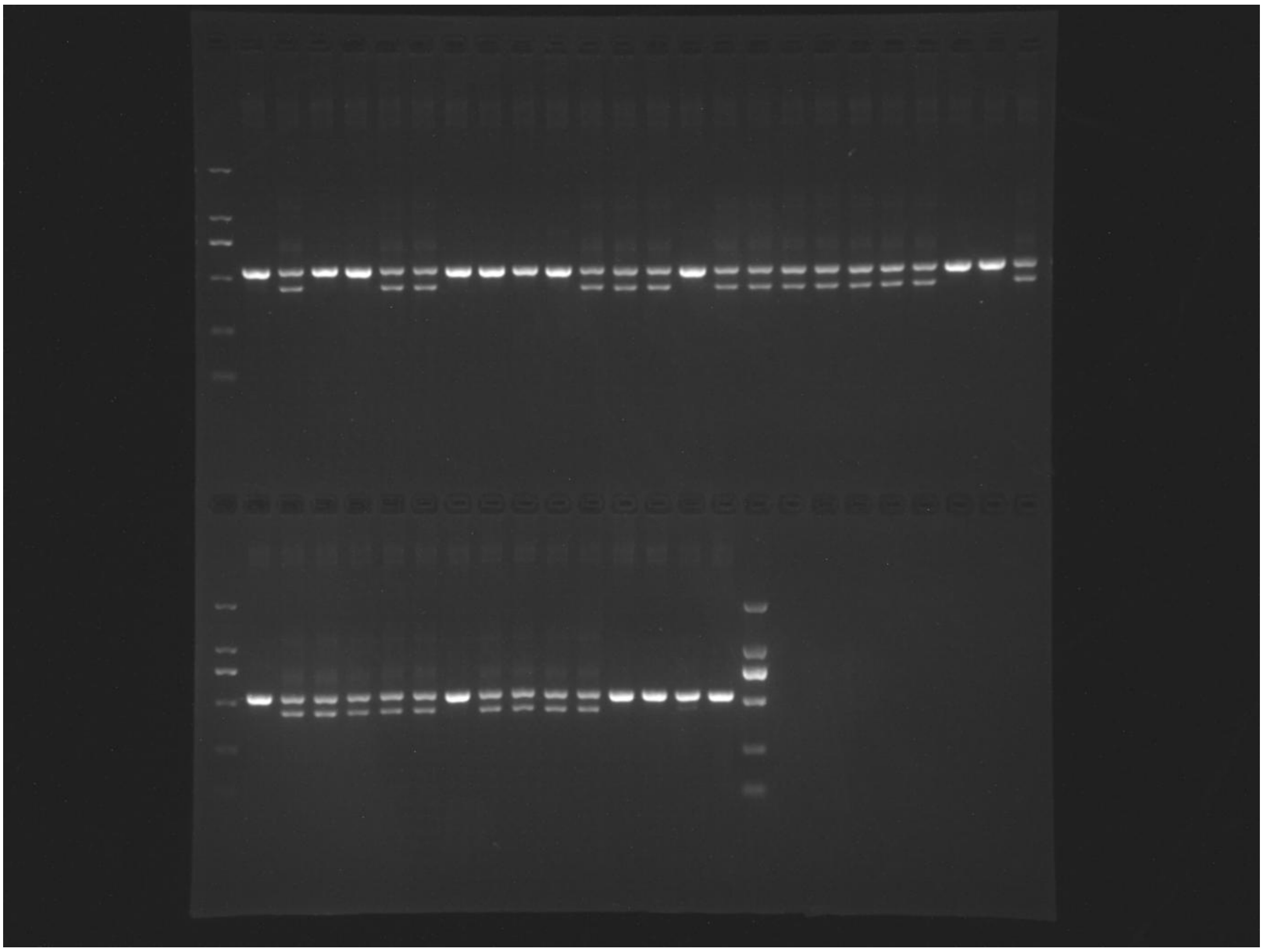

Figure S6. Uncropped agarose gel image corresponding to Figure 4C

Full, uncropped raw agarose gel image corresponding to the cropped panel shown in main-text Figure 4C. The region presented in Figure 4C is located in the upper-left portion of this gel. Molecular weight markers (DL2000 DNA Ladder; bands from bottom to top: 100, 250, 500, 750, 1000, 2000 bp) are present in three positions: on the left side of the gel and on the right side of the lower set of bands. The original gel image has not been edited.
